# Co-targeting EGFR and survivin with a bivalent aptamer-dual siRNA chimera effectively suppresses prostate cancer

**DOI:** 10.1038/srep30346

**Published:** 2016-07-26

**Authors:** Hong Yan Liu, Xiaolin Yu, Haitao Liu, Daqing Wu, Jin-Xiong She

**Affiliations:** 1 Center for Biotechnology and Genomic Medicine, Medical College of Georgia, Augusta University, Augusta, GA, 30912, USA; 2 Georgia Cancer Center at Augusta University, and Department of Biochemistry and Molecular Biology, Medical College of Georgia, Augusta University, Augusta, GA, 30912, USA.

## Abstract

Current targeted therapies using small kinase inhibitors and antibodies have limited efficacy in treating prostate cancer (PCa), a leading cause of cancer death in American men. We have developed a novel strategy by engineering an RNA-based aptamer-siRNA chimera, in which a bivalent aptamer specifically binds prostate-specific membrane antigen (PSMA) via an antibody-like structure to promote siRNA internalization in PCa cells, and two siRNAs specific to EGFR and survivin are fused between two aptamers. The chimera is able to inhibit EGFR and survivin simultaneously and induce apoptosis effectively *in vitro* and *in vivo*. In the C4-2 PCa xenograft model, the treatment with the chimera significantly suppresses tumor growth and angiogenesis. The inhibition of angiogenesis is mediated by an EGFR-HIF1α-VEGF-dependent mechanism. Our results support that the bivalent aptamer-driven delivery of two siRNAs could be a new combination therapeutic strategy to effectively inhibit multiple and conventionally “undruggable” targets.

Prostate cancer (PCa) is the most common cancer in American men, contributing to 220,800 new cases and 27,540 deaths in 2015^1^. Current therapies, although temporarily reducing cancer-related complications, do not have significant survival benefits. Particularly, single-agent treatment only exhibits limited activity in clinical settings, which may be attributed to the intrinsic and complex heterogeneity of a tumor^2^. Indeed, abnormalities in multiple tumor suppressors and oncogenes have been identified in PCa^3^, which may account for the failure of most targeted therapies that selectively block a single oncogenic molecule or signaling pathway. Moreover, the capability of cancer cells to escape a single-regimen therapy and switch to alternative survival signals highlights the importance of a combination approach to co-target multiple oncogenic pathways simultaneously.

Activation of EGFR signaling has been shown to increase cancer cell proliferation, enhance tumor vascularization and promote metastasis^4^,^5^. EGFR overexpression is associated with castration-resistant and high-risk PCa, as well as PCa bone metastasis^6^,^7^,^8^,^9^. EGFR inhibitors (e.g. erlotinib, gefitinib) have been used to treat prostate, pancreatic, lung, colorectal and head and neck cancers^10^,^11^. However, the benefit of EGFR inhibitors is temporary and can be quickly counteracted by acquired resistance^12^. Combination treatment, on the other hand, may restore the sensitivity of tumors to EGFR inhibitors. For example, the combined use of anti-MEK and anti-EGFR inhibitors can overcome the resistance of colorectal cancer^13^, and the combination of anti-EGFR and anti-VEGF agents have shown success and some have been approved for the clinical trials^14^.

Survivin, a member of the inhibitor of apoptosis (IAP) protein family^15^, plays a pivotal role in the progression of PCa and other solid tumors. Its overexpression has been correlated to recurrence, metastasis and therapeutic resistance^16^,^17^,^18^. Survivin has been actively pursued as an ideal target for cancer treatment. However, the portfolio of efficient survivin antagonists is small. Currently available inhibitors of survivin (such as YM155) have modest activity and are associated with side effects^19^. The lack of survivin-directed antagonists also reflects the limitation of current drug design, since only those molecules expressed on cell surface or having enzymatic activity are considered to be druggable. It remains challenging to discover small molecule inhibitors against cytoplasmic proteins (such as survivin).

The EGFR and survivin pathways represent two independent while interacting survival mechanisms in many cancer cells. As nodal proteins, EGFR and survivin intersect multiple signaling networks, therefore targeting both molecules might lead to global pathway inhibition regardless of tumor heterogeneity. Interestingly, it also has been shown that tumor resistant to EGFR inhibitors may switch to the survivin network for survival and recurrence^20^. Therefore, co-targeting EGFR and survivin may provide a novel strategy to more effectively inhibit multiple oncogenic signals. Current combination of kinase inhibitors has the overlapping toxicities, while monoclonal antibodies cannot access and block intracellular signaling molecules (e.g., survivin)^21^, and are usually associated with high costs, complex production and immunogenicity^22^.

Small interfering RNA (siRNA) has great potential for sequence-specific silencing of any genes and has emerged as a promising new therapeutic paradigm for “undruggable” targets^23^,^24^,^25^. However, the use of siRNA as a therapeutic has been hampered by the difficulty of delivery^26^. Recently, aptamers (synthetic DNA/RNA ligands) have proven to be a promising platform for delivering siRNA into cells. Selected in a process known as SELEX (systematic evolution of ligands by exponential enrichment)^27^,^28^, aptamers can specifically bind to various targets including organics, peptides, proteins and cells^29^. Particularly, cell-based SELEX allows the selection of internalized aptamers, which can induce the intracellular delivery of cargo through receptor-mediated endocytosis^30^. Aptamers have specific 3-dimentional structures for target binding with high affinity, which can be maintained *in vivo*. Aptamer-siRNA chimera (AsiC), employing only RNA molecules, is a new targeting therapeutic^31^,^32^ and has shown the promise of minimizing off-target effects that are usually associated with small molecule drugs and immunogenicity of antibody-based therapeutic. As a single-component entity, AsiC also has advantages in ease of synthesis and high tissue penetrability. Importantly, AsiC-based drugs can utilize endogenous enzymes (e.g. dicer, argonaute) and enable cell type- and mRNA sequence-specific gene silencing, which can provide selective and effective inhibition of protein targets regardless their cellular localization. For examples, CD4 aptamer-tat/rev siRNA chimera has shown the efficacy in inhibition of HIV transmission^33^, PSMA aptamer-PLK1siRNA enables the regression of prostate cancer^34^. CTLA4 aptamer-STAT3 siRNA inhibits tumor-associated Tregs and reduces tumor burden in multiple mouse tumor models^35^. EpCAM aptamer-survivin siRNA enables reversal of doxorubicin resistance and prolongs survival in mice bearing chemoresistant tumors^32^.

Currently, most chimeras are designed as the fusion of one aptamer with one siRNA^34^,^35^,^36^,^37^,^38^, and simultaneous delivery of multiple siRNAs has not been reported. Here, we have developed a novel chimera to co-deliver two siRNAs against EGFR and survivin via an efficient bivalent aptamer specific to prostate-specific membrane antigen (PSMA), a surface protein expressed on most PCa cells. The chimera can be processed by RNA interference machinery^39^. Inspired by an individual microRNA targeting multiple mRNAs^40^, we designed a chimera to target two mRNAs. The new chimera with a bivalent aptamer and two tandem siRNAs can simultaneously silence EGFR and survivin *in vitro* and *in vivo*, and demonstrates a profound efficacy against PCa growth through the induction of apoptosis and inhibition of angiogenesis. Co-delivery of two siRNAs in one chimera represents a novel approach for combination therapy of using siRNA molecules. Our design has the potential as a platform technology for advancing RNAi therapeutics.

## Results

### Engineering of a bivalent aptamer-dual siRNA chimera

#### PSMA aptamer-Survivin siRNA-EGFR siRNA-PSMA aptamer (PSEP)

**Figure 1:**
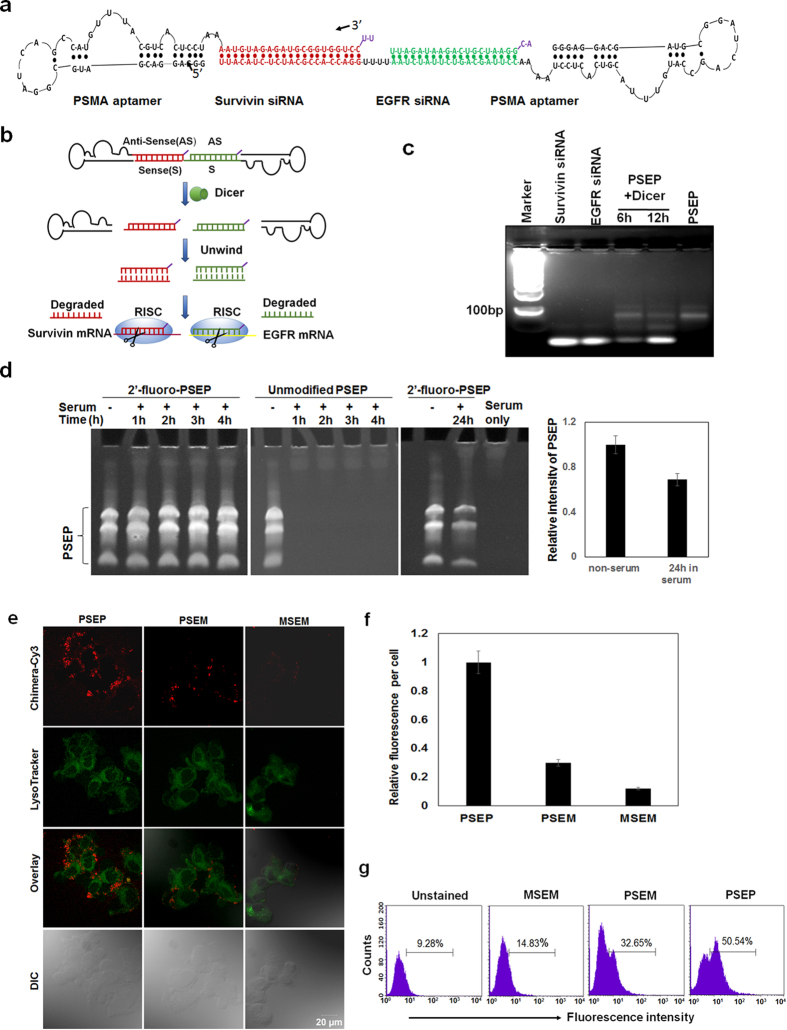
Design and characterization of bivalent aptamer-dual siRNA chimera. (**a**) Structure of PSMA aptamer-survivin siRNA-EGFR siRNA-PSMA aptamer (PSEP). PSEP chimera consists of a bivalent PSMA aptamer and two siRNAs specific to survivin and EGFR, respectively. Each antisense strand of siRNAs has a 2-nt overhang at the 3′ end. (**b**) Proposed mechanism of gene silencing. Upon internalization, PSEP chimera is recognized by Dicer. Dicer will process chimera into 21-nt siRNA duplex intermediates. The duplexes are unwound and recruited to the RNA-induced silencing complex (RISC) where Ago proteins mediate targeted mRNA silencing. (**c**) Dicer assay. PSEP chimera was treated with human recombinant dicer for 6 h or 12 h. The digestion patterns were resolved with 3.5% agarose gel electrophoresis. The gel images showed that the small siRNA was produced after PSEP was treated with dicer. The cropped gel is used in the main figures, and its full- length gel is presented in Supplementary Figure 14. (**d**) Evaluation of serum stability by denaturing 5% acrylamide/8 M urea gel electrophoresis. Unmodified or 2′F-modified PSEP was incubated with PBS buffer containing 50% human serum for 1–4 h, and 2′F-modified PSEP was incubated with PBS containing 50% human serum for 24 h. The cropped gels are displayed in the main figures. The full-length gels are presented in Supplementary Figure 15. PSEP intensity was measured with ImageJ. (**e**) Comparison of internalization of bivalent aptamer chimera vs monovalent control. Cy3-labeled PSEP, PSEM or MSEM were added into C4-2 cells for 2 h at 37 °C. LysoTracker Green was used to show lysosomes and endosomes. Confocal laser scanning microscopy was performed to show cell binding and internalization. Scale bar, 20 μm. (**f**) Quantification of the binding and internalization from confocal microscopy by ImageJ. 30–50 cells for each chimera are evaluated. **P < 0.01. (**g**) Detection of internalization. C4-2 cells were treated with Cy3-labeled PSEP, PSEM or MSEM for 2 h at 37 °C. Cells were washed with DPBS plus 0.5 M NaCl to remove surface bound RNAs. The amount of fluorescently labeled chimeras that internalized into cells was measured using flow cytometry.

In previous studies, we constructed PSMA aptamer-GFP siRNA chimera to silence the expression of GFP gene^41^. To increase the specificity, binding avidity and internalization of aptamer-siRNA chimera, we further designed a bivalent aptamer to facilitate the engagement of cell surface proteins and trigger cell activation^42^, thereby enhancing cargo internalization^43^. Bivalent aptamer is expected to have antibody-like properties in terms of inducing cell activation by cross-linking two receptors. PSMA aptamer against prostate-specific membrane antigen (PSMA) has been proven to effectively deliver siRNA into PSMA-expressing PCa cells^44^,^45^. Since an individual microRNA can target many different mRNAs, we design a new RNA chimera by fusing two siRNAs into the stem region of a chimera. We selected siRNAs against two important oncogenes: EGFR and survivin, respectively. First, three individual RNA molecules were prepared respectively, two of them contain a 39-nt PSMA aptamer (A10-3.2)^34^ and one strand of siRNAs. Specifically, one RNA is composed of a PSMA aptamer and a survivin anti-sense strand, and another RNA is composed of a PSMA aptamer and two tandem sense strands of siRNAs specific to EGFR and survivin. Two aptamer-containing RNA molecules are synthesized by T7 RNA polymerase-driven transcription with DNA template from PCR products. The third RNA, EGFR anti-sense stand, is synthesized by transcription as well. To unite the three RNAs to one, two aptamer-containing RNA molecules and EGFR anti-sense strand are mixed at the molar ratio 1:1:1, heated to 95 °C for 3 min, and cooled down slowly to the room temperature within 1 h. By annealing, a chimera with a bivalent aptamer and dual siRNAs is formed (Fig. 1a). We have designed survivin anti-sense and EGFR anti-sense strands with 2-nt overhang at the 3′ end of siRNA, which can facilitate the siRNA-RISC (RNA-induced silencing complex) formation^46^. To minimize nuclease-mediated degradation, 2′fluoro (F)-pyrimidines are incorporated into entire chimera via *in vitro* transcription. The inclusion of 2′F modification of all pyrimidines is expected to have improved serum stability of PSEP. To retain the flexibility and functionality of the aptamers and siRNAs, four “A” s are inserted between aptamer and siRNA, and four “U”s are inserted at the junction site of two siRNAs.

The mechanism of processing chimera is proposed and shown in Fig. 1b. PSEP chimera will enter to the cytoplasm and is processed by endonuclease dicer. Dicer will digest stem-loop containing PESP and produce two 21-nt siRNA duplex. After enzymatic unwinding siRNA duplex, anti-sense strand (guide strand) of siRNA will be selectively loaded into RISC complex, where Argonaute (Ago) protein family will mediate cleavage of mRNAs that are complementary to the siRNA guide strands. The sense strand (passenger) of siRNA will be degraded by endonucleases. To prove that PSEP can be effectively processed by dicer, we treated PSEP with human recombinant dicer for 6 h or 12 h. The digestion patterns were examined with 3.5% agarose electrophoresis. The gel images showed that the small RNAs were produced, with the same size as the free siRNAs against EGFR and survivin, suggesting PSEP can be processed by dicer (Fig. 1c).

### PSEP serum stability

We incubated PSEP chimera in phosphate-buffered saline (PBS) containing 50% of fresh human serum for 1, 2, 3 and 4 h. Denaturing 5% acrylamide/8 M urea gel electrophoresis revealed that 2′ F-modified PSEP did not show detectable degradation within 4 h; on the contrary, a degradation pattern was observed for unmodified PSEP in which no bands appeared as early as at 1 h. Over 60% of modified RNA kept the integrity (tight band) without degradation even after 24-h incubation (Fig. 1d). A similar result was also visualized by using fresh 50% mouse serum (Supplementary Figure 3). In agreement with our results, other studies have demonstrated that chemical modification can significantly enhance the resistance of RNA to nuclease attack^47^,^48^. In particular, replacing 2′-OH of RNA with 2′-amino or 2′-fluoro enhances the resistance to ribonucleases because ribonucleases select 2′-OH group for cleavage of phosphodiester bonds^49^. The results suggest that PSEP is stable in the serum.

### Comparison of internalization

Next, we compared the binding and internalization between monovalent versus bivalent chimeras with confocal fluorescence microscopy. An RNA aptamer specific to small organic dye Malachite Green (MG)^50^ with similar size and composition as A10-3.2 aptamer was selected as a non-targeting control. The chimera MSEM (MG aptamer-survivin siRNA-EGFR siRNA-MG aptamer) was constructed with the same length as PSEP. At the same time, the chimera PSEM (PSMA aptamer-survivin siRNA-EGFR siRNA-MG aptamer) which contains a functional PSMA aptamer and a negative control aptamer was established as a monovalent chimera control bearing the same size as PSEP. The one strand (aptamer-survivin antisense) of chimeras were labeled with Cy3-CTP during transcription and used to treat C4-2 cells, a PSMA-positive PCa cell line, for 2 h at 37 °C. At the same time, LysoTracker (spectrally distinguishable green fluorescence) was added into culture medium for exhibiting endosomes and lysosomes. As shown in Fig. 1e, the density of bivalent PSEP internalized into the cytoplasm within 2 h is significantly higher than that of monovalent PSEM, and non-targeting MSEM. PSEM mainly resides on the membrane surface, in contrast, bivalent-PSMA aptamer chimera is able to broadly distribute inside cytoplasm with some around the nucleus. The fluorescence intensity of each chimera was evaluated by ImageJ. The results (Fig. 1f) showed that PSEP treated-cell contained about 2-fold increased fluorescence than PSEM, and about 7-fold increased fluorescence than non-targeting MSEM.

Furthermore, quantitative flow cytometry was performed to compare the internalization. C4-2 cells were treated with Cy3-labeled chimeras followed by 0.5 M NaCl-DPBS washing to remove surface bound chimeras. The amount of internalized chimeras was quantitated using flow cytometry. As shown in Fig. 1g, the fluorescence intensity in PSEP treated C4-2 cells have increased about 1-fold compared with that in PSEM-treated cells, after subtracting nonspecific MSEM binding. Taken together, these results confirm the advantage of bivalent aptamer over the monovalent counterpart in siRNA internalization.

### PSEP chimera-mediated cell type-specific knockdown of target genes

**Figure 2:**
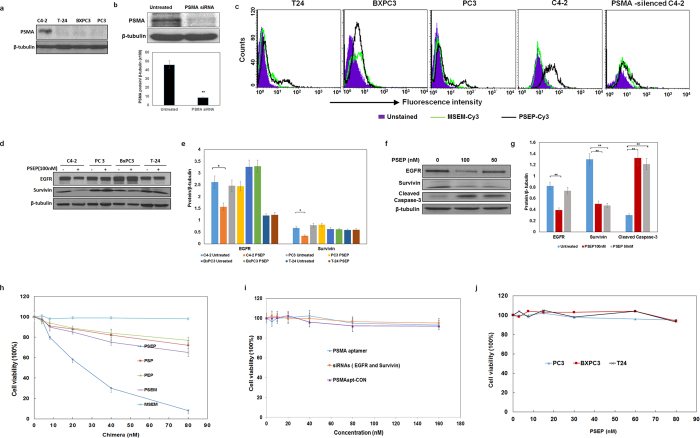
Characterization of PSEP on cell-specific binding, gene knockdown, and cytotoxicity. (**a**) Evaluation of PSMA expression by Western blot. The cropped blots are displayed in the main figures, the black lines surrounding blots indicate the cropping lines. The scanned full blots are presented in Supplementary Figure 16. (**b**). Knockdown of PSMA using siRNA and qualification of PSMA expression. Data are mean ± SEM (n = 3). **P < 0.01. The cropped blots are displayed in the main figures, the black lines surrounding blots indicate the cropping lines. The scanned full blots are presented in Supplementary Figure 17. (**c**) Cell binding assay by flow cytometry. C4-2, PC3, BXPC3 and T-24 cells were incubated with Cy3-labeled PSEP and Cy3-labeled MSEM, and detected by flow cytometry. Unstained cells are shown in solid blue, MSEM staining cells are shown in green line, and PSEP staining cells are shown in black line. (**d**) Detection of knockdown of EGFR and survivin by Western blot. The cropped blots are displayed in the main figures, and the black lines surrounding blots indicate the cropping lines. The scanned full blots are presented in Supplementary Figure 18. (**e**) Quantification of EGFR and survivin protein levels normalized by β-tubulin. The results are the mean ± SEM from three independent experiments. *P < 0.05. (**f**) Detection of cleaved Caspase-3 by Western blot. The cropped blots are displayed in the main figures. The scanned full blots are presented in Supplementary Figure 19. (**g**) Quantification of Western blot (**f**). Protein levels are normalized to β-tubulin. Data show mean ± SEM of three independent experiments. *P < 0.05, **P < 0.01. (**h**) Comparison of cytotoxicity of chimeras. C4-2 cells were treated with PSEP, PSP, PEP or MSEM at the varying concentrations. The results are representative of three independent experiments. (**i**) C4-2 cells were treated with PSMA aptamer alone, simply mixed siRNAs specific to survivin and EGFR, and PSMAapt-CON (scrambled siRNA). The results are representative of three independent experiments. (**j**) Cell type-specific cytotoxicity assay. Cell lines were treated with the varying concentrations of PSEP for 72 h. The results are representative of three independent experiments.

Four cell lines were examined on the expression of PSMA with Western blot. Three cancer cell lines including PC3 (prostate), BXPC3 (pancreatic) and T-24 (bladder) are negative for PSMA, while C4-2 cells have strong PSMA expression (Fig. 2a). Furthermore, we evaluated the binding specificity of PSEP chimera on different cell lines. The flow cytometry demonstrated that PSEP chimera has specific binding capability on PSMA-expressing C4-2 cells, but not PSMA-negative BXPC3, PC3 and T-24 cell lines (Fig. 2c). To further validate whether PSMA aptamer indeed binds to PSMA protein, we knocked down PSMA with PSMA siRNA, as shown in Fig. 2b. After 72 h transfection of PSMA siRNAs by lipofectamine RNAi MAX, Western blot was performed and proved the PSMA knockdown. Next, flow cytometry was performed to evaluate the aptamer binding. The result indicates that PSEP significantly reduces the staining for C4-2 cells (Fig. 2c), which confirms A10-3.2 aptamer possesses PSMA-specific binding capability. Next, cell lines were treated with PSEP for 72 h, and expression levels of EGFR and survivin were determined by Western blot. As shown in Fig. 2d,e, in PSMA-expressing C4-2 cells, the protein levels of EGFR and survivin have been significantly reduced compared with the untreated C4-2 cells. In contrast, there are no detectable suppression of EGFR and survivin in PSMA-negative cells lines including PC3, BXPC3 and T-24. The results indicate that PSEP, but not MSEM, MG aptamer or scrambled siRNA control (Supplementary Figures 4 and 5), is able to co-deliver two siRNAs and concomitantly silence two target genes in a cell type-specific manner. Furthermore, PSEP treatment resulted in a dose-dependent increase in cleaved capase-3, an indicator of apoptosis^51^ (Fig. 2f,g). The results suggest that the silencing of EGFR and survivin by PSEP is associated with activated apoptotic signaling in PSMA-positive cancer cells.

Next, we have evaluated the silencing efficacy of siRNAs in each construct by lipofectamine transfection. Conventional lipofectamine-based transfection is independent of aptamers and allows to compare the native silencing efficacy of siRNAs. Gene silencing of EGFR and survivin in PSMA positive C4-2 and PSMA-negative PC3 cells were evaluated with Western blot. In C4-2 cells, as shown in Supplementary Figure 7a,b, the siRNAs in each chimera have well preserved their native silencing efficacy compared with EGFR siRNA and survivin siRNA. Transfected PSEP can silence 80–90% of EGFR and 85–95% of survivin, while PSEP without lipofectamine can silence 40–50% of EGFR and 50–60% of survivin. Chimeras of PSEM, PSEM and MSEM showed the very similar efficacy in silencing EGFR (80–90%) and survivin (85–95%) by lipofectamine transfection. In PC3 cells, PSEP without lipofectamine did not silence EGFR or survivin, suggesting cell specificity of PSEP. By lipofectamine transfection, all constructs have achieved gene knockdown at similar levels to the native EGFR siRNA and survivin siRNA (Supplementary Figure 8a,b). In lipofectamine transfection experiments, all constructs are free labeled and present with native structures. We have compared that PSEP with or without 2′-fluororine modification showed the very similar silencing efficacy by lipofectamine transfection (Supplementary Figure 6).

### PSEP chimera-mediated cell type -specific cytotoxicity

To assess cell viability in the presence of PSEP, PSEM, MSEM, PEP and PSP, C4-2 cell lines were exposed to chimeras for 72 h at the varying concentrations (Fig. 2h). At the concentration of 80 nM, the reductions in C4-2 cell viability induced by individual aptamers are: about 92% (PSEP), 35% (monovalent PSEM), 25% (PSP), 21% (PEP), and 1% (MSEM), respectively. IC_50_ of PSEP is about 26 nM. PSEP also showed the killing activity for PSMA positive LNCaP cells (Supplementary Figure 2). In a set of control experiment (Fig. 2i), C4-2 cells were treated with PSMA aptamer, or mixed siRNAs specific to EGFR and survivin, or PSMAapt-CON chimera without targeting any mRNA, respectively. C4-2 cells did not have reduced cell viability when exposed to chimera alone, simply mixed siRNAs, or control chimera. Our results suggest that PSMA aptamer alone does not have tumor killing activity, in agreement with previous studies^34^,^44^. Since siRNA cannot diffuse freely through cell membrane, it is well known the delivery systems are needed to aid siRNA function. Therefore, it is not surprising that simply mixed siRNAs without a carrier are ineffective for cell killing. Furthermore, three PSMA-negative cancer cell lines (PC3, BXPC3 and T-24) were treated with varying concentrations of PSEP. The result shows that three PSMA-negative cancer cell lines do not respond to the PSEP treatment and there was no significant changes in cell viability upon PSEP treatment (Fig. 2j). The viability assay indicates that PSEP possesses strong cell type-specific cytotoxicity, and specifically attacks PSMA-expressing PCa, suggesting potentially lower side effects *in vivo*.

Furthermore, cytotoxicity was evaluated after lipofectamine transfection of each constructs. The IC_50_ is about 11–13 nM for PSEP, MSEM and PSEM, 20 nM for PSP, and 40 nM for PEP, respectively (Supplementary Figure 9). As the controls, PSMA and PSMA-CON did not show any cytotoxicity, and the IC_50_ is 18 nM for survivin siRNA, 38 nM for EGFR siRNA, and 28 nM for equal molar mixed survivin & EGFR siRNAs, as shown in Supplementary Figure 10. For comparing cytotoxicity of PSEP in different cell lines, PC3, BXPC3 and T24 were treated with varying concentrations of PSEP. The IC_50_ is about 24 nM in PC3 cells, 41 nM in BXPC3 cells, while 74 nM in T24 cells, respectively, as shown in Supplementary Figure 11. These experiments confirm that siRNAs inserted into chimeras have well preserved silencing effect and potent cytotoxicity. PSEP without lipofectamine has less efficacy than transfected PSEP. The significantly different IC_50_ for transfected PSEP in different cell lines indicates the each tumor has different survival pathways.

### PSEP-induced time-dependent apoptosis in C4-2 cells

**Figure 3:**
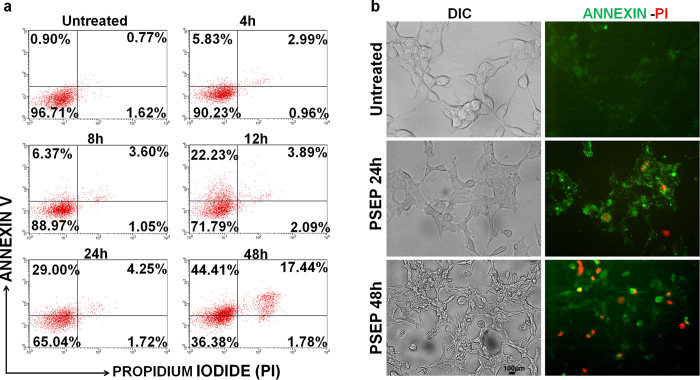
Detection of apoptosis with flow cytometry and fluorescence microscopy. C4-2 cells were treated with PSEP for the varying durations. (**a**) Flow cytometry showed the increased populations at early-apoptosis (Annexin V + PI^−^) and at late apoptosis (Annexin V + PI^+^). The stage movement confirmed that apoptosis occurs upon PSEP treatment. Consistently (**b**) epi-fluorescence microscopy showed apoptosis pattern: cell shrinkage and increased signals of Annexin V (green) and PI (red). Scale bar, 100 μm.

To further confirm apoptosis occurrence upon PSEP treatment, flow cytometer was performed to monitor the dynamic process of cell death. First, C4-2 cells were exposed to PSEP for different time durations (4–48 h). Apoptosis events were detected using Annexin V-Propidium Iodide (PI) staining. In a time-course measurement, as shown in Fig. 3a, after 4-h treatment, there was an increase in early apoptotic cell population (Annex V + PI^−^) from 0.90% without treatment to 5.83% with treatment, and an increase in the late apoptosis (Annex V + PI^+^) from 0.77% to 2.99%. After 12 h, entire population apparently moved toward to early apoptosis, quantitatively, 22.3% of cells are at the stage of early apoptosis, 3.89% at the stage of late apoptosis. After 24 h, 29% cells are at the early apoptosis and 4.25% at the late apoptosis. Up to 48 h, the early apoptotic cells increased to 44.41% and late apoptotic and dead cells increased to 17.44%. C4-2 cell population in the presence to PSEP progressed from viable, early apoptosis to the late apoptosis. This “stage movement’’ of C4-2 cells after PSEP treatment indicates that PSEP suppresses C4-2 cell survival through apoptosis, which is directly related to downregulation of EGFR and survivin. There were consistent changes in cell morphology and apoptotic pattern, as identified from fluorescence microscope imaging (Fig. 3b). Compared with untreated control, most cells have increased Annexin V signal (green) after 24 h treatment, and at 48 h, increased PI signal (red) were observed. Cell morphology showed shrinkage and optically dimmer compared with untreated control cells.

### Reduction of tumor burden and inhibition of tumor-associated neovasculature by systemic administration of PSEP

**Figure 4:**
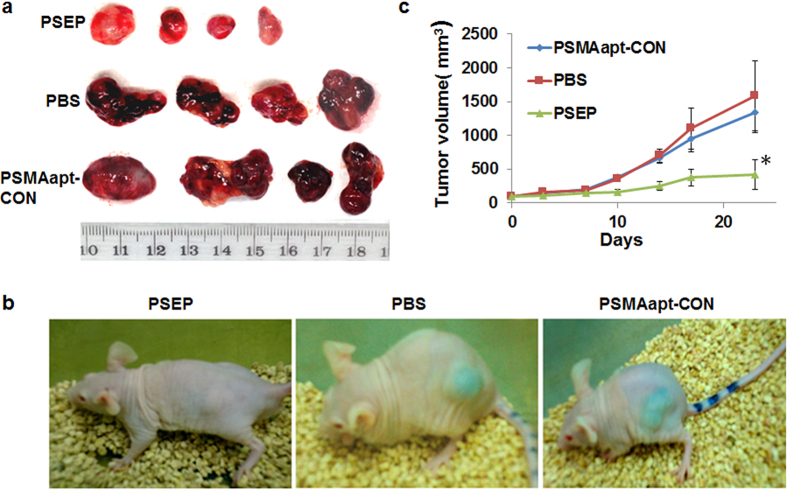
Systemic administration of PSEP significantly suppresses tumor growth and reduces tumor-associated angiogenesis. (**a**) Direct observation of the tumors after treatments with PBS, PSMAapt-CON and PSEP. PSEP treated tumors are significantly smaller than PBS- and PSMAapt-CON- treated tumors. Remarkably, in contrast to the dark and bloody tumors in control groups, tumors in PSEP group have changed to much less bloody with lighter color. (**b**) Representative tumor-bearing mouse imaging. With whole-body imaging, tumors on control mice (PBS and PSMAapt-CON) are blue, in contrast, tumors post PSEP treatment are grey and white under the skin. (**c**) Corresponding tumor growth curves. After C4-2 cells were implanted into athymic mice, PSEP, PBS or PSMAapt-CON was intraperitoneally injected to mice. Following the treatment, tumors were measured using a digital caliper twice a week. (n = 4, ^*^P < 0.05).

To assess the impact of PSEP on PCa growth *in vivo*, subcutaneous C4-2 xenografts were established in athymic nu/nu male mice. PSEP (100 μl, 20 μM) per mouse were injected intraperitoneally to tumor-bearing mice every other day for beginning 1 week and every day for the following two weeks. Control mice were injected intraperitoneally with equivalent volume of PBS or PSMAapt-CON (100 μl, 40 μM) (i.e. at the same moles of aptamer and siRNA as PSEP). Following a 21-day treatment, 3–4 fold reduction in tumor volume was observed upon treatment with PSEP, as compared with the tumors treated with PBS or non-silencing PSMAapt-CON (Fig. 4a,c). Notably, the color of C4-2 xenograft was significantly changed after treatment. In live mice, a clear blue color in the PBS-and PSMAapt-CON-treated mice has changed to a pale-to-white color in PSEP-treated mice (Fig. 4b). Consistently, from macroscopic observation, freshly dissected *ex-vivo* tumors showed visible difference in color and size after PSEP treatment versus those in the tumors treated with PBS and PSMAapt-CON. Tumors from PBS-and PSMAapt-CON-treated mice were dark, bloody and highly vascularized, in contrast, tumors from PSEP treated mice were pale and poorly vascularized. These results suggest that PSEP may have inhibitory effect on angiogenesis of C4-2 tumors. In another cohort animal experiment, we treated mice with MSEM (100 μl, 20 μM) or PBS (100 μl) every other day for 7 days followed by injection every day for 14 days. Using tumor size as the indicator, no significant efficacy was observed upon the treatment with MSEM chimera (Supplementary Figure 12).

### Assessment of anti-angiogenesis effect of PSEP

**Figure 5:**
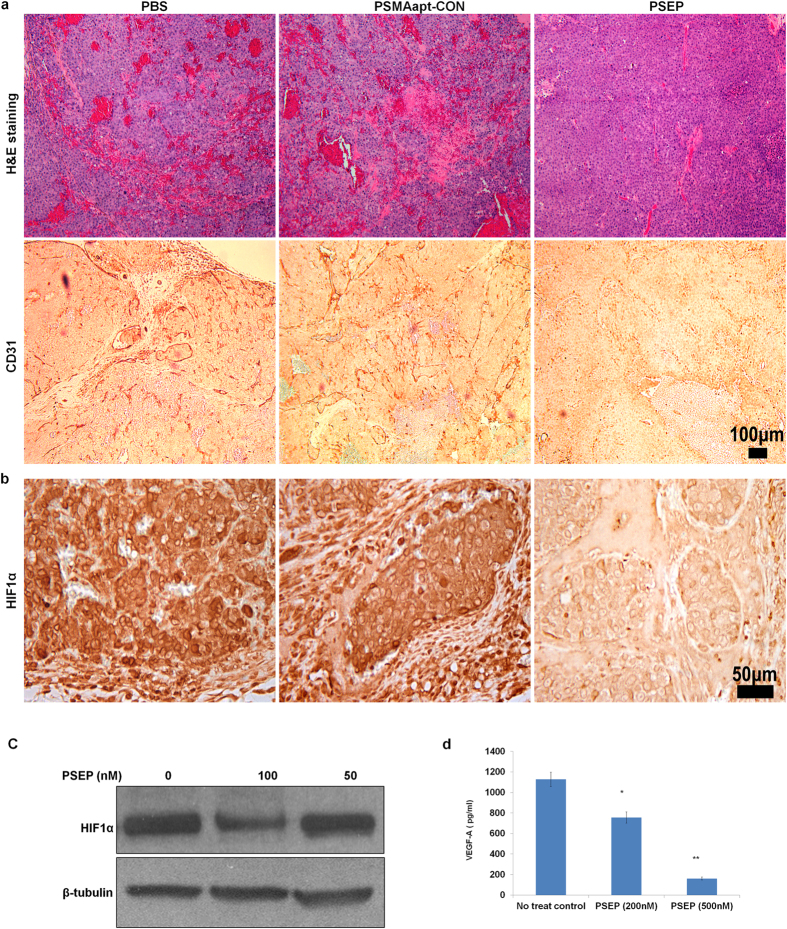
Histology analysis of tumors and identification of anti-angiogenesis effect of PSEP. (**a**) H&E staining and CD31 IHC to identify blood vessels. H&E staining exhibited that tumor tissues in controls (PBS and PSMAapt-CON) are enriched with high-density blood vessels, which span entire tumors, in contrast, after PSEP treatment, tumors have much less density of blood vessels. IHC assay for CD31 expression further revealed that blood vessel density of tumors has been significantly reduced upon PSEP treatment. Furthermore, detection of HIF1a expression at tissue (**b**) and at cultured cells (**c**). IHC staining of tumor tissues revealed the significant decrease of HIF1α *in vivo*, and Western blot showed that the reduction of HIF1α in C4-2 cells was observed after PSEP treatment. The cropped blots are displayed in the main figures, and the black lines surrounding blots indicate the cropping lines. The scanned full blots are presented in Supplementary Figure 20. (**d**) Detection of VEGF-A secretion from C4-2 cells. C4-2 cells were treated with PSEP for 72 h. The culture supernatants were measured for VEGF-A secretion by ELISA. PSEP can significantly inhibit the expression of VEGF- A in C4-2 cells at a dose-dependent manner. *P < 0.05, and **P < 0.01.

To confirm the anti-angiogenesis effect of PSEP, histology analysis was performed. Remarkably, H&E staining revealed that the blood vessel density has been significantly reduced after PSEP treatment compared with controls treated with PBS or PSMAapt-CON. The high-density blood vessels span the entire tumors in control mice, consistent with the observed blue color of these control tumors, whereas PSEP-treated tumors show much less blood vessels, consistent with the observed much lighter color of PSEP-treated tumors. CD31 immunohistochemistry (IHC) was performed to further demonstrate the change of microvessels after PSEP treatment. C4-2 tumors from PBS and PSMAapt-CON groups have densely distributed blood vessels; remarkably, a significant reduction in CD31-stained blood vessels was observed after administration of PSEP (Fig. 5a). The results suggest that PSEP is able to inhibit angiogenesis.

Tumor angiogenisis heavily depends on the growth factors released from tumor cells, since the growth factors will target on endothelial cells and stimulate the growth of host blood vessels. C4-2 cells are able to secret high level of VEGF-A^52^, which contributes to the development of vascularized tumors in animal models.To determine whether PSEP inhibits angiogenesis through a VEGF-dependent mechanism, C4-2 cells were treated with PSEP for 72 h, the cuture supernatants were colleceted and analyzed with VEGF-A ELISA kit. VEGF-A in untreated controls is 1100 ± 70 pg/ml, and reduced to 755 ± 55 pg/ml (decrease by 31%) upon 200 nM PSEP treatment, and was further reduced to 161 ± 15 pg/ml (decrease by 85%) upon 500 nM PSEP treament (Fig. 5d). We further investigated the mecahnism by which PSEP inhibits VEGF angiogenesis. It has been reported that EGFR promotes the expression of VEGF and activates autocrine VEGF signaling in endothelia cells^53^. In squamous cell carcinoma, it has been shown that EGFR inhibitors (gefitinb and erlotinb) decrease VEGF expression via Hypoxia-inducible factor 1-alpha (HIF1α)^54^, a direct upstream regulator for VEGF transcription^55^. To evaluated if EGFR silencing has affected on HIF1a, IHC was employed to detect HIF1a in tumor tissues and ELISA was used to detect HIF1α in cultured C4-2 cells. Consistently, both methods confirmed the decrease of HIF1a after PSEP treatment (Fig. 5b,c). Taken together, these results suggest that the anti-angiognesis effect of PSEP is, at least partially, mediated through an EGFR-HIF1α-VEGF pathway.

### Gene silencing and tumor cell apoptosis *in vivo*

**Figure 6:**
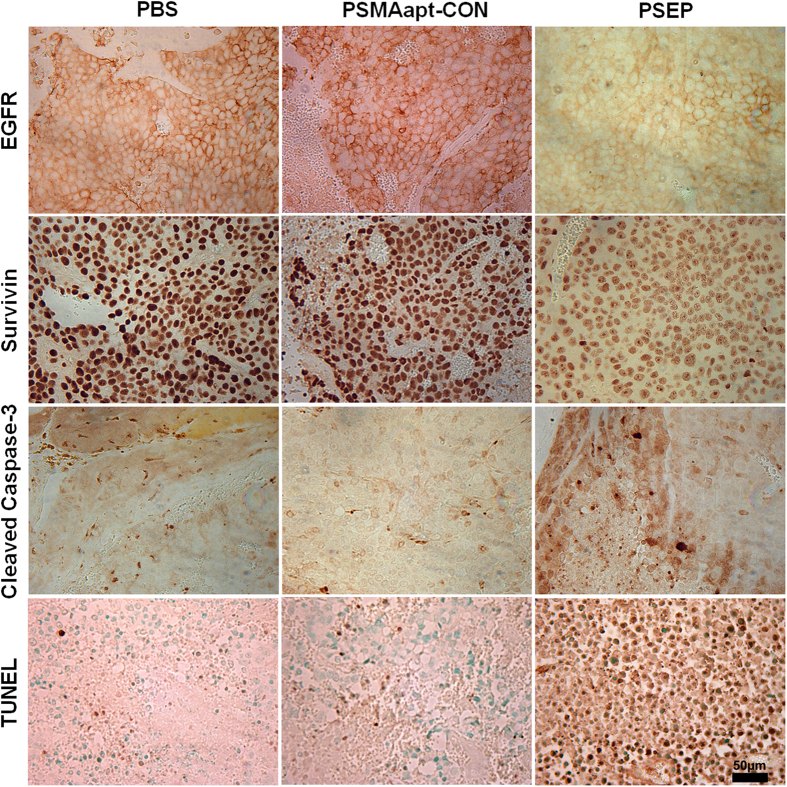
Evaluation of gene expression and apoptosis after PSEP treatment *in vivo*. Formalin-fixed paraffin-embedded sections of xenograft tumors were stained with antibodies targeting EGFR, survivin, cleaved caspase-3. TUNEL assay was performed to detect the apoptosis-associated DNA damage. Comparing with PBS and PSMAapt-CON, PSEP treatment has significantly reduced the expression of EGFR and survivin, and significantly up-regulated cleaved Caspase -3. TUNEL assay further revealed the much stronger DNA damage in PSEP treatment tumors than that in controls. Scale bar, 50 μm.

Next, we evaluated gene regulation and apoptosis *in vivo*. As shown in Fig. 6, PSEP treamtment has significantly inhibited the expression of EGFR and survivin compared with PBS and non-silencing PSMAapt-CON, and consistently, dramatically upregulated cleaved formed Caspase-3. Furthermore, TUNEL (terminal deoxynucleotidyl transferase-mediated dUTP nick end-labeling) was performed to *in situ* detection of apoptosis-triggered DNA fragmentation in tumor tissue, which represents a characteristic hallmark of apoptosis. PSEP treated tumors have much stronger TUNEL staining than that of PBS- and PSMAapt-CON-treated tumors. IHC staining also demonstrated that PSEP can significantly increase P21 and reduce Ki67 (Supplementary Figure 1). The histology results indicate that PSEP enables the knockdown of EGFR and survivin and induces tumor cell apoptosis *in vivo*, which is translated into a significant suppression of tumor growth in xenograft prostate cancer.

### Immunogenicity and toxicity

**Figure 7:**
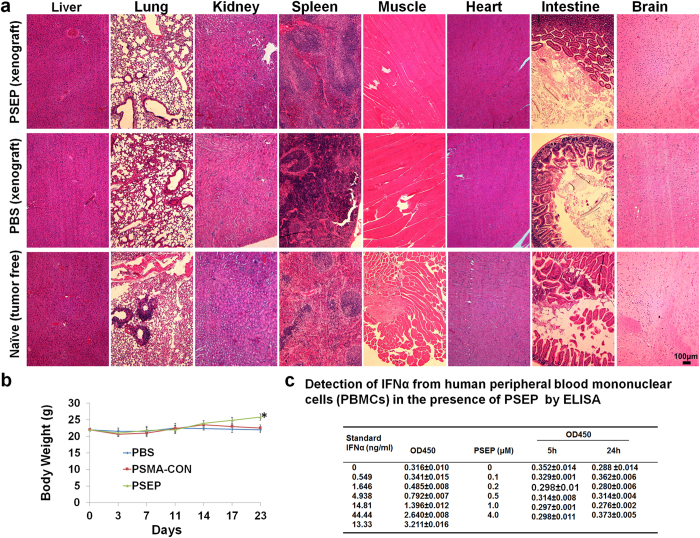
Immunogenicity and toxicity. (**a**) Histological evaluation of the tissue damages after treatment. The organs (heart, lung, liver, kidney, brain, muscle, spleen, and intestine) were removed for H& E staining. Compared with naïve (no tumor implant), the organs from PSEP treated xenografts do not exhibit significant histological change. (**b**) Following PSEP treatment, mouse body weights were monitored. PSEP treated mice have a significant increase of body weight. (**c**) Detection of interferon response. IFNα from normal human peripheral blood mononuclear cells upon treatment with PSEP was measured. The expression of in culture supernatants were quantified with human IFNα ELISA Kit. There was no detectable IFNα in the test range from 100 nM up to 4 μM, which represents 8 folds as high as the dose used in the experiments.

We have examined the histopathology of major tissues of xenografts after administering PSEP, or equivalent volume of PBS as well as naïve tumor-free mice. As shown in Fig. 7a, H&E staining shows that there are no differences in major organs among PSEP-, PBS- treated or naïve mice. The body weights in the PSEP treated group were higher than controls (PBS and PSMAapt-CON) (Fig. 7b). The body weight gain reflects the global improvement after PSEP treatment, and it clearly has no acute toxicity. It was reported that siRNA or siRNA/carriers can stimulate sequence-dependent innate immune responses associated with interferon-α (IFN-α)^56^. Therefore, we measured IFN-α releasing using human peripheral blood mononuclear cells (PBMCs) following PSEP challenge for 5 h and 24 h. As shown in Fig. 7c, no postive IFNα signals are detactable in PBMCs. We also measured serum IFN-α and IL-6 between PBS- and PSEP-treated mice. As shown in Supplementary Figure 13, all IFN-α levels from PBS-and PSEP-treated mouse sera were undetectable and within the background level, and IL-6 between PBS and treatment mice did not show statistical difference. These results suggest that PSEP RNA chimera does not trigger innate immune response and also does not have acute toxicity.

### 5-RACE (rapid amplification of cDNA ends) detection of PSEP directed gene silencing through RNAi pathway

**Figure 8:**
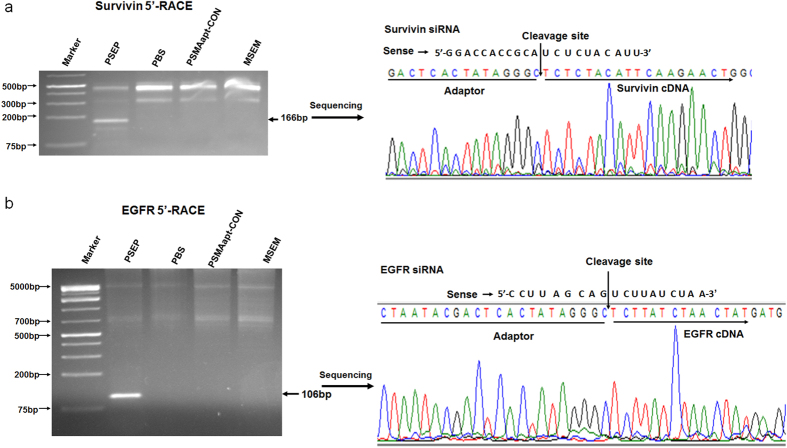
5′-RACE PCR assay to evaluate PSEP mediated gene silencing through RNAi pathway. Tumor RNA was extracted and transcribed into cDNA with a SMARTerIIA oligonucleotide adaptor. Nested PCR was performed to amplify gene products specific to EGFR and survivin. PCR products with expected sizes were sequenced. The cropped gel is used in the main figures, and its full-length gel is presented in Supplementary Figure 21.

Tumor RNAs were isolated and reverse transcribed into cDNA with an adaptor. By using nested PCR, the expected 166 bp of survivin and 106 bp of EGFR PCR products were resolved and visualized in agarose gels. Sequencing of PCR products confirm that cleavages present on the 10-nt from 5′-end of antisense strands of EGFR and survivin siRNAs as shown in Fig. 8. Therefore, PSEP mediated gene knockdown is through RNAi pathway.

## Discussion

Heterogeneity is an intrisic characteristic of human cancer, particularly at advanced stages. Combination therapy to target serveral oncogenic pathways simultaneously, therefore, may have better efficacy in retarding or eradicating tumors. Small molecule drug combination usually shows some efficacy initially, but reaches plateu with increased toxicity and quickly developed drug resistance. For example, although current kinase inhibitor combinations show the efficacy and certain targeting, most kinase inhibitors tend to target on multiple kinases (low specificity), and combination of different kinases may more easily cause overlapping toxicities. Combination of monoclonal antibodies are usually more specific but have limitations in antagonizing intracellular targets/signalling and high immunogenicity due to their membrane impermeability and recongnition by host as foreign.

Aptamer-siRNA chimera, built with only RNA molecules, is an attractive therapeutic strategy for its potential low immunogenicity, low production cost, ease of chemical modification and modularity. In this investigation, we have developed a new aptamer-siRNA chimera which consists of two siRNAs and a bivalent PSMA aptamer. We demonstrated that the resulting PSEP chimera specifically and effectively suppresses the endogenous expression of EGFR and survivin in PCa cells expressing PSMA, and significantly inhibits tumor growth in mouse models.

The structure of PSEP chimera can be processed by RNAi machinary. Bivalent aptamer offers much enhanced cargo internalization and target specificity. Similarly, the strategies by increasing ligand valency to promote cargo delivery are validated to be efficient in nanoparticle-based carriers^57^. Two tandem siRNAs spaced with four “U” are designed to warrant the cleavage with dicer but not mixing two genes, since dicer is able to measure and cut 21–25-nt RNA duplex^39^,^58^,^59^. Meanwhile, we have examined the sequence complementarity among PSMA aptamer, survivin siRNA and EGFR siRNA, and no significant complementary sequences are found, ensuring the correct folding by annealing. The size of PSEP is about 59Kd, which is much larger than current PSMA aptamer-siRNA chimeras^34^,^60^ (around 29Kd). To increase the *in vivo* circulation half-life, 20Kd PEG has been added to the chimera in the previous study^34^ and proven to be effective. Chimera with a bivalent aptamer and dual siRNAs should offer increased circulating half-life and reduced renal excretion.

In this construct, we have incorporated 2′F into entire RNA chimera by T7 RNA polymerase -driven transcription. Previous reported aptamer-siRNA chimeras contain one strand unmodified siRNA^34^,^36^. Our 2′F completely modified RNA should offer more serum stability than partial modified chimeras. The efficacy in tumor targeting and gene knockdown of both EGFR and survivin was confirmed, and the profound reduction of tumor size and inhibition of tumor-associated blood vessels have been achieved, suggesting the efficacy of targeting on multiple proliferation pathways.

Tumor angiogenesis is regulated by multiple mechanisms. Among them, VEGF expressed by tumor cells has been shown to play an essentail role. PESP specifically targets tumor-associated vessels as suggested by the reduction of blood vessels. PSEP can significantly inhibit VEGF secretion from C4-2 cells. We further demonstrated that the inhibition of VEGF by PSEP was at least partially due to the blockade of EGFR-HIF1α signaling, which has been shown to be capable of inducing VEGF expression. Indeed, PSEP significantly reduced EGFR and HIF1*α* at both cellular and tissue levels. We envsion that the effecacy of PSEP on anti-angiogensis will contribute to many vascularized tumors since PSMA expression is up-regulated on endothelial cells of tumor-associated neovasculature, but not on normal endothelia cells^61^,^62^.

Our work is a proof-of-concept study indicating that two different siRNAs can be simultaneously delivered by a bivalent aptamer. Co-delivery of two siRNAs in one RNA chimera provides a new and efficient approach for combination therapy. Since the system is highly modular, our work can be applied to many targeting co-delivery design by using siRNA and aptamer. Bivalent aptamer offers high specificity and affinity, although previous studies used bivalent aptamer for siRNA delivery *in vitro*^60^, this is first report to prove bivalent aptamer functionality *in vivo*. Our data also demonstrated that repeated administration is well tolerated and did not elicite innate immune response. For further clinical translation, immunogenicity and toxicity should be examined with higher species of animal models. The pharmaokinetics and optimization of doses and the injection time intervals also need to be investigated.

In summary, we have developed a PSMA-specific chimera capable of efficiently delivering and silencing two genes *in vivo*. The modularity of this construct opens a way to design various combination treatments with a wide array of siRNAs and appearing cell-specific aptamers. For its high tumor specificity, low immunogenicity and ease of production, PSEP chimera can be a promising gene therapy to improve clinical outcomes for prostate cancer.

## Materials and Methods

### Chemicals and Cell culture

Vendors for specific chemicals are listed below. Cell culture products were purchased from Invitrogen (Carlsbad, CA). Antibodies were from Cell Signaling Technology (Danvers, MA) except for anti-CD31 from Abcam (Cambridge, MA) and PSMA from BioLegend (San Diego, CA). Single stranded DNAs were synthesized by Integrated DNA Technologies (IDT, Coralville, IA). TranscriptAid T7 High Yield Transcription Kits and Cy3-CTP were purchased from Thermo Fisher Scientific. PCR reagents were from Sigma-Aldrich (St Louis, MO). LysoTracker Green DND-26 and Alexa Fluor 488 Annexin V/Dead Cell Apoptosis kits were from Invitrogen. ELISA kits for detection of VEGF-A, IFNα and IL-6 were obtained from RayBiotech (Norcross, GA). TUNEL assay kit was purchased from R&D systems (Minneapolis, MN). Cell lines including PC3, BXPC3 and T-24 were purchased from the American Type Culture Collection (ATCC, Manassas, VA) and C4-2 cells were from Dr. Daqing Wu’s Laboratory. SMARTer RACE 5′/3′ kits were purchased from Clontech (Mountain View, CA). ELISA kit for mouse IFN alpha was obtained from R&D systems. GeneSolution siRNA specific to PSMA was ordered from Qiagen (Germantown, MD). Human serum (Normal Pool) was obtained from Thermo Fisher Scientific.

### Mouse

All animal studies were approved by the Institutional Animal Care and Use Committee at Georgia Regents University. Athymic nu/nu mice were purchased from Harlan Laboratories, Inc. The methods were carried out in accordance with the approved guidelines.

#### Aptamer-siRNA Chimera synthesis

The ssDNA templates and primers were synthesized from IDT.

**For PSEP** (PSMA aptamer-surivin siRNA-EGFR siRNA-PSMA aptamer), three RNA molecules (RNA1, RNA2 and RNA3) were constructed individually:

**RNA 1**: PSMA aptamer- survivin antisense siRNA: 5′-GGGAGGACGAUGCGGAUCAGCCAUGUUUACGU CACUCCUAAAAUGUAGAGAUGCGGUGGUCCUU-3′.

RNA1 PCR template: 5′-GGGAGGACGATGCGGATCAGCCATGTTTACGTCACTCCTAAAATGTAGAG ATGCGGTGGTCCTT-3′.

RNA1 5′primer: 5′-**TAATACGACTCACTATA**GGGAGGACGATGCGG-3′ (**F1)**. The forward primer contains T7 RNA polymerase promoter site (bolded).

RNA1 3′primer: 5′-AAGGACCACCGCATCTCTACATTTTAGGAGTGACGTAAAC-3′ (**R1**)

**RNA2**: PSMA aptamer-EGFR sense siRNA-survivn sense siRNA: 5′-GGGAGGACGAUGCGGAUCAGCCAUGUUUACGUCACUCCUAAAAC CUUAGCAGUCUUAUCUAAUUUUGGACCACCGCAUCUCUACAUU-3′.

RNA2 PCR template: 5′-GGGAGGACGATGCGGATCAGCC ATGTTTACGTCACTCCTAAAACCTTAG CAGTCTTATC TAATTTTGGACCACCGCATCTCTACATT-3′

RNA2 5′primer: F1

RNA2 3′primer: 5′-AATGTAGAGATGCG GTGGTCCAAAATTAGA-3′ (**R2**)

**RNA3:** EGFR anti-sense strand: 5′-UUAGAUAAGACUGCUAAGGCA-3′.

RNA3 PCR template: 5′-TAATACGACTCACTATATTAGATAAGACTGCTAAGGCA-3′

RNA3 5′primer: 5′-TAATACGACTCACTA-3′ (**F2**)

RNA3 3′primer: 5′-TGCCTTAGCAGTCTT-3′ (**R3**).

Three RNAs were generated by *in vitro* transcription with PCR products as templates. The PCR products were sequenced or put into T-A cloning pCR2.1 vector (Invitrogen) and sequenced. Transcription was performed with TranscriptAid T7 High Yield Transcription Kit following manufacture’s instruction. 2′F-modified pyrimidines (TriLink, San Diego, CA) were incorporated into RNA to replace CTP and UTP. In some cases, the chimeras were synthesized with a Cy3-labeled CTP. The transcribed RNAs were purified with phenol/chloroform/isoamyl alcohol (25:24:1) (Sigma-Aldrich), precipitated with isopropanol (Sigma-Aldrich) followed by cold 70% ethanol wash. The RNA pellets were dissolved in nuclease free water (IDT).The purification procedures were used for all transcribed RNAs. Three RNAs were mixed at molar ratio 1:1:1 and annealed to form one entity by heated at 94 °C for 3 min followed by slowly cooling to room temperature within 1 h.

**For PSMAapt-CON** (PSMA aptamer-scrambled siRNA): 5′-GGGAGGACGAUGCGGAUCAGCCAUGUUU ACGUCACUCCUAAAAAACAGUCGCGUUUGCGACUGG-3′. Two RNAs (RNA4 and RNA5) were synthesized individually.

**RNA4:** PSMA aptamer- scrambled anti-sense siRNA: 5′-GGGAGGACGATGCGGATCAGCCATGTTTACG TCACTCCTAAAACCAGUCGCAAAGCGCUGACAC-3′

RNA4 PCR template: 5′-GGG AGGACGATGCGGATCAGCCATGTTTACGTCACTCCTAAAA-3′,

RNA4 5′primer: F1,

RNA4 3′primer: 5′-TTGTCAGCGCTTTGCGACTGGTTTTAGGAGTGACGTAAAC-3′ (**R4**)

**RNA 5**: Scrambled siRNA sense strand: 5′-GTGTCAGCGCUUUGCGACUGG-3′

RNA5 PCR template: 5′-TAATACGACTCACTATAGTGTCAGCGCTTTGCGACTGG-3′

RNA5 5′primer: 5′-TAATACGACTCACTA-3′ (**F3**)

RNA5 3′primer: 5′-CCAGTCGCAAAGCGCT-3′ (**R5**)

RNA4 and RNA5 were generated with transcription and annealed at molar ratio of 1:1 to generate PSMAapt-CON chimera.

For **PSP** (PSMA aptamer-survivin siRNA-PSMA aptamer). Two RNAs (RNA1 and RNA6) are individually constructed and annealed to form PSP. RNA1 (PSMA aptamer-survivin antisense siRNA) was described above.

**RNA 6**: PSMA aptamer- survivin sense siRNA: 5′GGGAGGACGAUGCGGAUCAGCCAUGUUUACGUCA CUCCUUUGGACCACCGCAUCUCUACAUU-3′.

RNA6 PCR template: 5′-GGGAGGACGATGCGGATCAGCCATGTTTACGTCACTCCTTTGGACCACCG CATCTCTACATT-3′.

RNA6 5′primer: F1

RNA6 3′primer: 5′-AATGTAGAGATGCGGTGGTCCAAAGGAGTGACGTAAACATG-3′ (**R6**)

**For PEP** (PSMA aptamer-EGFR siRNA-PSMA aptamer), two RNAs (RNA7 and RNA8) were individually constructed and annealed to form PEP.

**RNA 7:** PSMA aptamer-EGFR antisense siRNA: 5′-GGGAGGACGAUGCGGAUCAGCCAUGUUUAC GUC ACUCCUAAAAUUAGAUAAGACUGCUAAGGCA-3′

RNA7 PCR template: 5′-TAATACGACTCACTATAGGGAGGACGATGCGGATCAGCCATGTTTACGTC ACTCCTAAAATTAGATAAGACTGCTAAGGCA-3′.

RNA7 5′primer: F1.

RNA7 3′primer: 5′-TGCCTTAGCAGTCTTATCTAATTTTAGGAGTGACGTAAAC-3′ (**R7**)

**RNA8:** PSMA aptamer-EGFR sense siRNA: 5′-GGGAGGACGAUGCGG AUCAGCCAUGUUU ACGU CA CGUCCUCCUUAGCAGUCUUAUCUAAUU-3′

RNA8 PCR template: 5′-GGGAGGACGATGCGGATCAGCCATGTTTACGTCACGTCCTCCTTAGCA GTCTTATCTAATT-3′.

RNA8 5′primer: F1.

RNA8 3′primer: 5′-AATTAGATAAGACTGCTAAGGAGGACGTGACGT-3′ (**R8**)

**For MSEM**: MG aptamer (specific to Malachite Green)-survivin siRNA-EGFR siRNA-MG aptamer, three RNA molecules (RNA3, RNA9 and RNA10) were individually constructed and annealed together to form MSEM. RNA3 (EGFR anti-sense strand) was described above.

**RNA9**: MG aptamer-EGFR sense siRNA-survivn sense siRNA: 5′-GGAUCCCGACUGGCGAGAGCCAGGU ACGAAUGGAUCCAAAAACCUUAGCAGUCUUAUCUAAUUUUGGACCACCGCAUCUCUACAUU-3′

RNA9 PCR template: 5′-GGATCCCGACTGGCGAGAGCCAGGTAACG AATGGATCCAAAAACCTTAGC AGTCTTATCTAATTTTGGACCA CCGCATCTCTACATT-3′

RNA9 5′primer: 5′-TAATACGACTCACTATAGGATCCCGACTGGCGAGAGCCAGG-3′ (**F4**)

RNA9 3′primer: 5′-AATGTAGAGATGCGGTGGTCCAAAATTAGA-3′ (**R9**)

**RNA10**: MG aptamer-survivin antisense siRNA: 5-GGAUCCCGACUGGCGAGAGCCAGGUAACGAA UG GAUCCUU UUGUAGAGAUGCGGUGGUCCUU-3′

RNA10 PCR template: 5′-GGATCCCGACTGGCGAGAGCCAGGTAACGAATGGATCCTTTTGTAGA GATGCGGTGGTCCTT-3′

RNA10 5′primer: F4

RNA10 3′primer: 5′-AAGGACCACCGCATCTCTACAAAAGGATCCA-3′ (**R10**)

**For PSEM** (PSMA aptamer-survivin siRNA-EGFR siRNA-MG aptamer), three RNAs (RNA9, RNA1 and RNA3) were annealed together at the molar ratio of 1:1:1.

### *In vitro* dicer assay

PSEP (4 μg) was digested using human recombinant dicer enzyme (2 units) at 37 °C for either 6 h or 12 h following manufacturer’s instructions (Genlantis, San Diego, CA). Reaction was quenched by adding dicer stop solution. The digestion pattern was analyzed on 3.5% agarose gel electrophoresis.

### Serum stability assay

2′F-modified and unmodified PSEP (2 nmol) were incubated with final 50% human serum at 37 °C for 1–4 h. In another test, 2′F-modified PSEP (2 nmol) were incubated with 50% human serum for 24 h. RNA integrity was detected with denaturing 5% acrylamide/8 M urea gel electrophoresis^63^. PSEP intensity was measured with ImageJ (NIH).

### Evaluation of binding and internalization by confocal microscopy and flow cytometry

C4-2 cells were seeded on 12 mm (diameter) cover-glass at a density of 5 × 10^4^ cell/well for 24 h in RPMI 1640 supplemented with 5% fetal bovine serum. Cy3-labeled PSEP, PSEM, or MSEM (100 nM) was added into culture for 2 h at 37 °C. At the same time LysoTracker Green DND-26 (80 nM) and yeast tRNA (300 μg/ml) was added to the culture medium for imaging. Images were captured using confocal laser scanning microscope (Zeiss LSM 510) and analyzed with Zeiss LSM image Browser Version 4.0. Quantification of Cy3 fluorescence intensity is through ImageJ (NIH).

Quantitative flow cytometry was performed to detect internalized chimeras. C4-2 cells (1 × 10^5^/well) in 6-well culture plates were cultured for 24 h in RPMI 1640 supplemented with 5% fetal bovine serum. Cy3-labeled PSEP, PSEM, or MSEM (100 nM each) was added into culture for 2 h at 37 °C. Yeast tRNA (300 μg/ml) was added to the medium during culture for blocking nonspecific binding. Next, cells were washed with DPBS plus 0.5 M NaCl to remove surface bound RNAs^34^. The internalized chimeras were detected with BD FACSCalibur flow cytometry.

### Cytotoxicity assay

Cellular cytotoxicity was quantified by measuring WST-8 formazan using Cell Counting Kit-8 (CCK-8) (Dojindo, Japan). Cells in RPMI 1640 containing 5% fetal bovine serum were seeded in 96-well plate at a density of 5 × 10^3^ in 5% CO_2_ incubator for 24 h at 37 °C. Cell lines of C4-2, PC3, BXPC3 and T24 were incubated with the varying concentrations of PSEP for 72 h without transfection reagents (e.g., Lipofectamine). In the control experiment, C4-2 were incubated with different concentrations of constructs at quadruplicate wells of each concentration point for 72 h. CCK-8 solution (10 μl) was added to each well and incubated at 37 °C for 4 h. Absorbance at 450 nm was measured using a plate reader.

### Western blot

Cells were lysed in lysis buffer (M-PER Mammalian Protein Extraction Reagent, Thermo Fisher Scientific) containing 1x Halt Protease Inhibitor Cocktails. The cell lysates were kept on ice for 40 min and vortexed for 3 times and centrifuged at 12,000 × g for 10 min at 4 °C. The supernatant was collected and the protein concentration was determined with Bio-Rad Protein Assay (Bio-Rad, Hercules, CA). Protein (100 μg) was mixed with 2x Laemmli sample buffer containing 5% *β*-mercaptoethanol and heated at 95 °C for 10 min. Denatured samples was separated on 10% SDS-polyacrylamide gel and transferred to PVDF membrane. The membranes were blocked with 5% non-fat milk overnight at 4 °C, and then incubated with primary antibodies for 2 h at room temperature, followed by incubation with horseradish peroxidase-conjugated secondary antibodies for 2 h at room temperature. After ECL Western Blotting Substrate (Pierce) was added onto membrane, the signals were captured by the exposure to X-ray film. Western blot was quantified using ImageJ (NIH).

### Cell type specific binding assay

C4-2, PC3, BXPC3 and T24 cells were trypsinized and washed with PBS. After washing, cells were incubated with Cy3-labeled PSEP (50 nM) or Cy3-labeled MSEM (50 nM) in the presence of yeast tRNA (300 μg/ml) for 30 min at 37 °C. Cell binding were detected using BD FACSCalibur flow cytometry.

### PSMA knockdown

C4-2 cells were plated in 6-well plates at a density of 2 × 10^5^ cells/well for 24 h. PSMA siRNAs targeting on 4 sequences of PSMA gene (30 pmol each) was transfected into cells using Lipofectamine RNAi MAX (Invitrogen) according to the manufacturer’s instruction. Cells were harvested 72 h post-transfection. PSMA knockdown was detected by Western blot analysis. PSMA silenced cells were used for aptamer binding assay using BD FACSCalibur flow cytometry.

### Detection of apoptosis by flow cytometry and fluorescent microscopy

C4-2 prostate cancer cells were seeded onto cover glass for florescence imaging and into 12-well plates for flow cytometry. C4-2 cells (2 × 10^6^) were treated with PSEP (100 nM) for different time durations. The cells were harvested and washed in cold phosphate-buffered saline (PBS). Cells were stained with Alexa Fluor 488 Annexin V- Propidium Iodide (PI) solution for 15 min at room temperature. For imaging, fluorescence microscopy (Nikon Eclipse TE2000-S) was used to capture each channel signals separately and merged with ImageJ Plugin for colocalization. For flow cytometry, cells (1 × 10^4^/sample) were acquired by BD FACSCalibur and analyzed using BD FACStation software.

### Xenograft models

4 to 5-week-old male athymic nu/nu mice were injected subcutaneously with C4-2 cells (2 × 10^6^) mixed with matrigel (v/v 1:1) (Corning, NY) at the left flank of mice. Upon tumor reaching 100 mm^3^, mice were randomly divided into three groups. PSEP (100 μl, 20 μM)) or PSMA-CON (100 μl, 40 μM), or MSEM (100 μl, 40 μM) or PBS (100 μl) was intraperitoneally injected into the mice every other day for 7 days and followed by injection every day for 14 days. Tumor sizes and body weights were measured twice a week. The tumor volume was calculated according to the formula: V = (L × W^2^)/2 (W, the width; L, length). The animals were euthanized two days after the last treatment. The tumors and organs (liver, spleen, kidney, brain, heart, muscle, blood and intestine) were removed and fixed in 10% formalin buffer. The sections of tissues were analyzed by hematoxylin and eosin (H&E) staining and immunohistochemistry.

### VEGF assay

C4-2 cells were seeded into 24-well plates at the density of 1 × 10^6^/well for 24 hours in the 5% CO_2_ incubator at 37 °C. The culture medium was changed to serum free. The PSEP with the varying concentrations were added into the culture. After 72-h incubation, the cell culture supernatants were collected. The VEGF-A in supernatants was determined by human VEGF-A ELISA kit following the manufacture’s instruction.

### IFNα assay

Normal human peripheral blood mononuclear cells (PBMCs) were separated with BD Vacutainer cell preparation tubes with sodium citrate. Cells were seeded into 24-well plates at 10^6^/well for 24 h in RPMI medium containing 10% fetal bovine serum. PSEP with the varying concentrations was added into cells, and cells were incubated for 5 h and 24 h in 5% CO_2_ incubator at 37 °C. The cell culture supernatant was detected with human IFNα ELISA kit following the manufacture’s instruction. Mouse blood was collected from facial vein and separated to get serum by centrifugation for 10 min at 13,000 rpm. Serum IFNα from PSEP- or PBS-treated mice were analyzed using mouse IFNα kit.

### Histology assay

Animals were euthanized with CO_2_, and tumors and organs (spleen, lung, kidney, intestine, heart, liver and brain) were removed and fixed with 4% paraformaldehyde in 0.1 M sodium phosphate buffer (PH 7.6). Tissues were cut into 3 mm sections and were dehydrated in graded series of alcohol and xylene, and embedded in paraffin. Sections (6 μm) were cut and mounted on the slides, deparaffinized in xylene and ethyl alcohol. Each block has a section for H&E staining. For immunohistochemistry assay, sections were incubated in 3% normal goat serum for 2 h and followed by overnight incubation with primary antibodies:caspase-3 (1:20), survivin (1:800), EGFR (1:50), HIF1α (1:100), Ki67 (1:100), P21(1:100) and CD31 (1:25). After washing, the sections were incubated with biotinylated secondary antibody (1:200, VECTOR, Burlingame, CA) for 1 hour. Following washing, the sections were incubated with VECTASTAIN ABC reagents for 30 min. The immunoreactivity (IR) was visualized with the substrate solution (VECTOR). The images were captured with Nuance fluorescence microscope with bright field imaging system. TUNEL assay was performed according to the manufacturer’s instruction. Paraffin embedded tissues were sectioned, dewaxed, hydrated and digested with Proteinase K. After washing, slides were immersed into quenching solution for 5 min, then incubated with TdT labeling buffer for 1 h in a humidity chamber. Slides were washed in PBS and incubated with streptavidin-HRP for 10 min. After washing, DAB work solution was added into the slides. Following washing, slides were counterstained with Methyl Green. The images were captured with Nuance fluorescence microscope.

### 5′-rapid amplification of cDNA ends (5′-RACE) PCR analysis

5′-RACE was performed using SMARTer RACE5′/3′ kit according to the manufacturer’s protocol. RNA (3 μg each) from tumors treated with different chimeras was reverse transcribed into cDNA containing a SMARTerIIA oligonucleotide adaptor. Nested PCR was performed to detect the cleavage sites. For EGFR siRNA analysis, outer PCR was first run with EGFR reverse primer (5′-GGGCAGGTGTCCTTGCACGT-3′) and forward Universal Primer A Mix (UPM) (5′-CTAATA CGACTCACTATAGGGCAAGCAGTGGTATCAACGCAGAGT-3′); then inner PCR was performed with EGFR reverse primer (5′-GCACGGCGCCATGCAGGATTTCCTGT-3′) and forward primer Universal Primer Short (5′-CTAATACGACTCACTATAGGGC-3′). For survivin analysis, outer PCR was first performed with survivin reverse primer (5′-TGCTAAGGGGCCCACAGGAAGGCTGGT-3′) and forward primer: UPM; then inner PCR was performed with survivin reverse primer (5′-AGCCTTCCAGCTCCTT GAAGCA-3′) and forward primer Universal Primer Short. PCR products were separated with 2% agarose gel electrophoresis, and DNA was extracted from gel with NucleoSpin Gel and PCR Clean-Up kit (Clontech). The purified PCR products were sequenced to determine identity.

### Statistical analysis

The results were expressed as a mean ± SD. All Data were analyzed using two-tailed Student’s t-test (Graph Pad Prism) by comparing with the control group, and P < 0.05 was considered statistically significant.

## Additional Information

**How to cite this article**: Liu, H. Y. *et al*. Co-targeting EGFR and survivin with a bivalent aptamer-dual siRNA chimera effectively suppresses prostate cancer. *Sci. Rep.* **6**, 30346; doi: 10.1038/srep30346 (2016).

## Supplementary Material

Supplementary Information
