# Supplementary material for: Co-targeting EGFR and survivin with a bivalent aptamer-dual siRNA chimera effectively suppresses prostate cancer
Source: Sci Rep. 2016 Jul 26;6:30346. doi: 10.1038/srep30346 (PMC4960556; doi:10.1038/srep30346)
Supplement: Supplementary Information [file srep30346-s1.pdf]

SUPPLEMENTARY INFORMATION

Co-targeting EGFR and survivin with a bivalent aptamer-dual siRNA chimera effectively suppresses prostate cancer

Hong Yan Liu[1*], Xiaolin Yu[1], Haitao Liu[1], Daqing Wu[2*] & Jin-Xiong She[1]

[1]Center for Biotechnology and Genomic Medicine, Medical College of Georgia, Augusta University, Augusta, GA, 30912; [2] Georgia Cancer Center at Augusta University, and Department of Biochemistry and Molecular Biology, Medical College of Georgia, Augusta University, Augusta, GA, 30912.

Correspondence and request for materials should be addressed to H.Y.L. (email: HOLIU@ augusta.edu) or D.W. (email: DWU@augusta.edu)

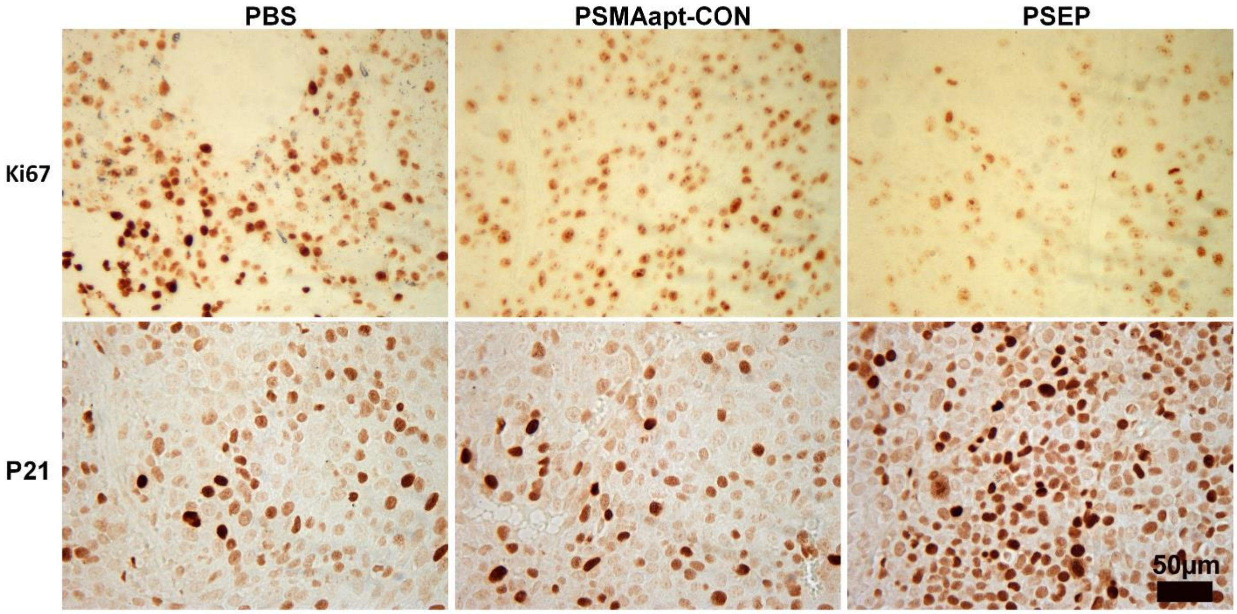

Supplementary Figure 1. Assessment of Ki67 and P21 after PSEP treatment. IHC staining was performed as described in Materials and Methods. Tumor expression of P21 is significantly increased upon PSEP treatment, as compared with that in PBS, or PSMAapt-CON treatment groups. It also demonstrates that PSEP is able to decrease Ki67 expression. Scale bar, 50μm.

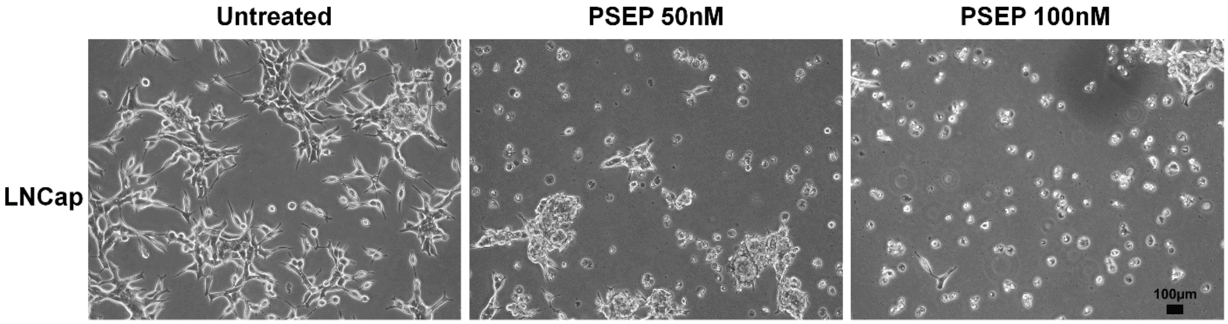

Supplementary Figure 2. Effect of PSEP on PSMA-expressing LNCaP cells. LNCaP cells were treated with PSEP at the concentrations of 50nM and 100nM for 48 h. Microscopic imaging shows detached and apoptotic cell morphology after being exposed to PSEP, indicating PSEP has significant killing activity. Scale bar, 100µm.

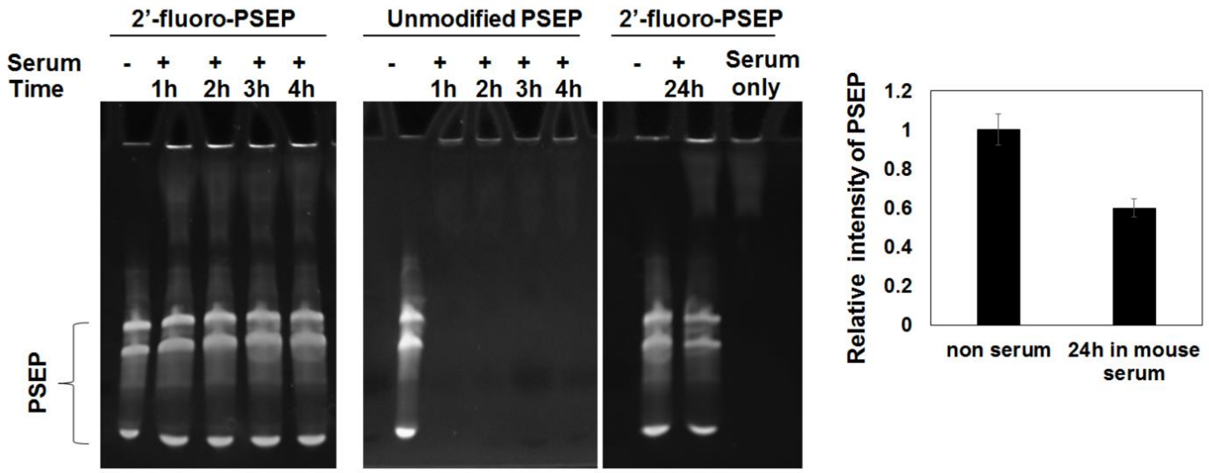

Supplementary Figure 3. The PSEP stability in 50% fresh mouse serum. 2'F-modified and unmodified PSEP (2nmol) were incubated with 50% mouse serum for 24 h. RNA integrity was detected with denaturing 5% acrylamide/8M urea gel electrophoresis. PSEP intensity was measured with ImageJ. 2'F-modified PSEP still has over 60% integrity after 24 h incubation.

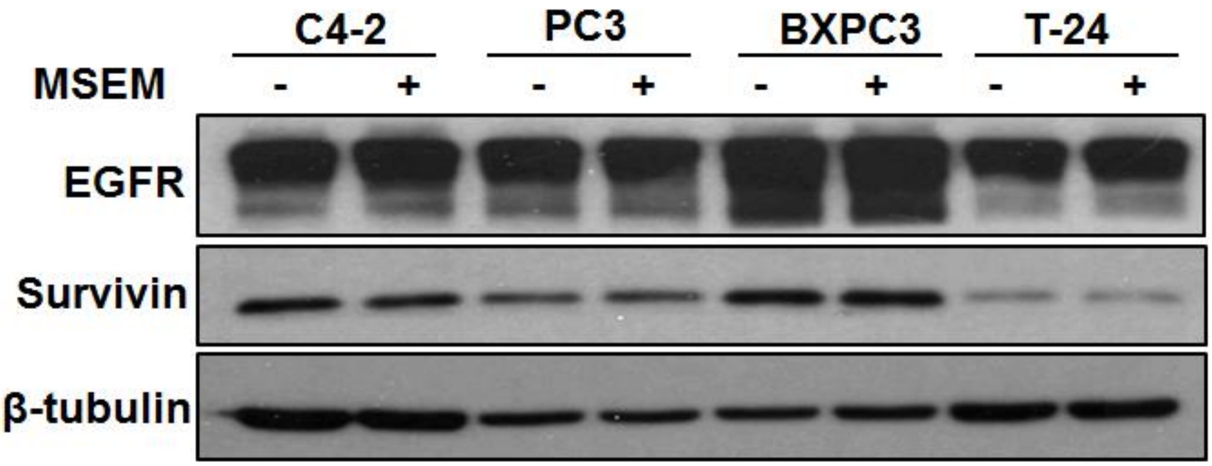

Supplementary Figure 4. Detection of knockdown of EGFR and survivin in the presence of MSEM. Different cell lines were treated with MSEM (100nM, 2'-F labeled) for 72h. EGFR and survivin were detected with Western blot. All cell lines including PSMA-expressing C4-2, PSMA- negative: PC3, BxPC3 and T-24 cells, did not show detectable changes in the expression of EGFR and survivin.

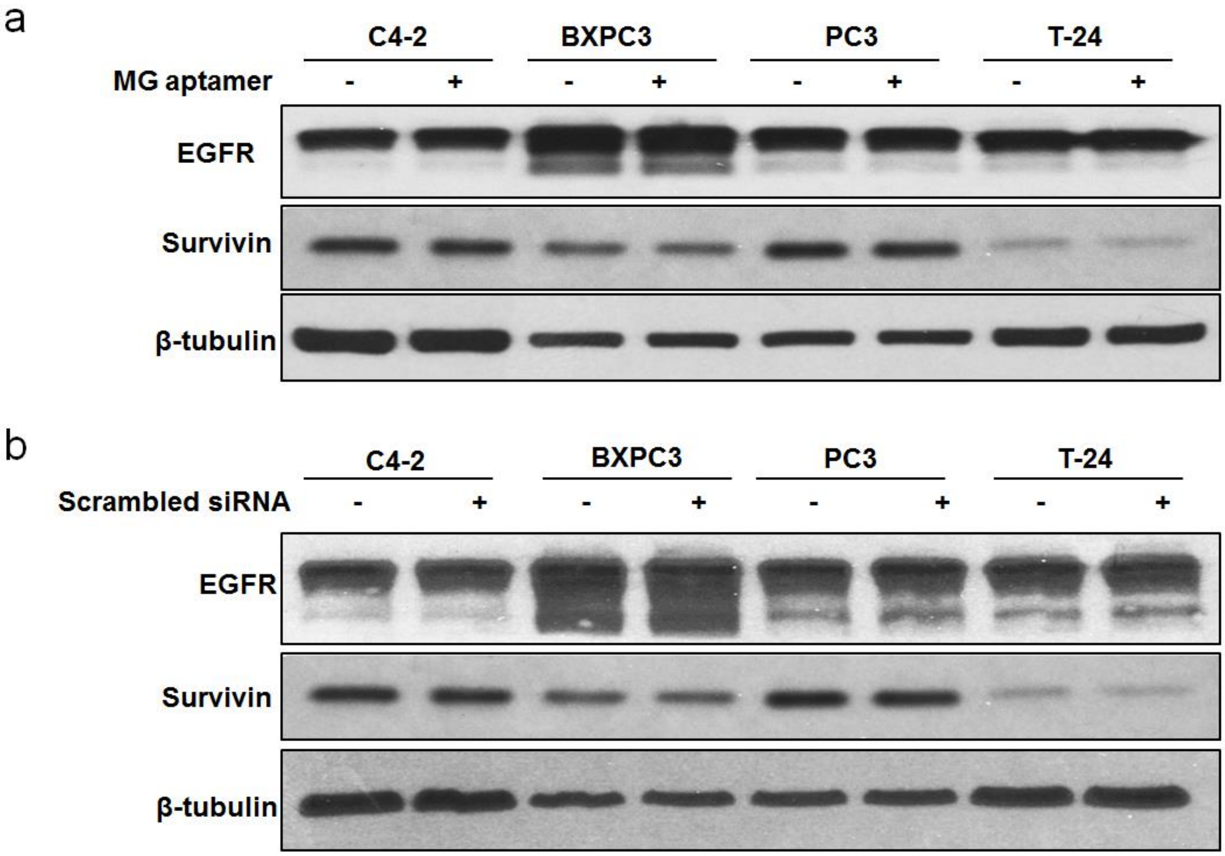

Supplementary Figure 5. Detection of knockdown of EGFR and survivin in the presence of MG aptamer or scrambled siRNA. Different cell lines were treated with MG aptamer or scrambled siRNA (2'-F labeled, 100nM) (Trilink, CA) for 72h. EGFR and survivin were detected with Western blot. (a) MG aptamer, and (b) scrambled siRNA. All cell lines including PSMA-expressing C4-2, PSMA-negative: PC3, BXPC3 and T-24 cells, did not show detectable changes in the expression of EGFR and survivin upon treated with MG aptamer or scrambled siRNA.

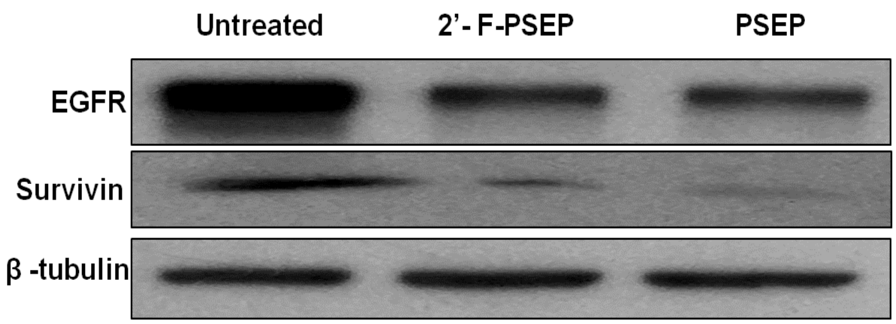

Supplementary Figure 6. Comparison of silencing efficacy of 2'-fluro-PSEP and PSEP using Lipofectamine RNAiMAX (Invitrogen). 2'-fluor labeled or non-labeled PSEP (50nM) were transfected into C4-2 for 72h. EGFR and survivin were evaluated with Western blot. The result did not show detectable difference in the expression of EGFR and survivin between 2'-fluor labeled and non-labeled PSEP.

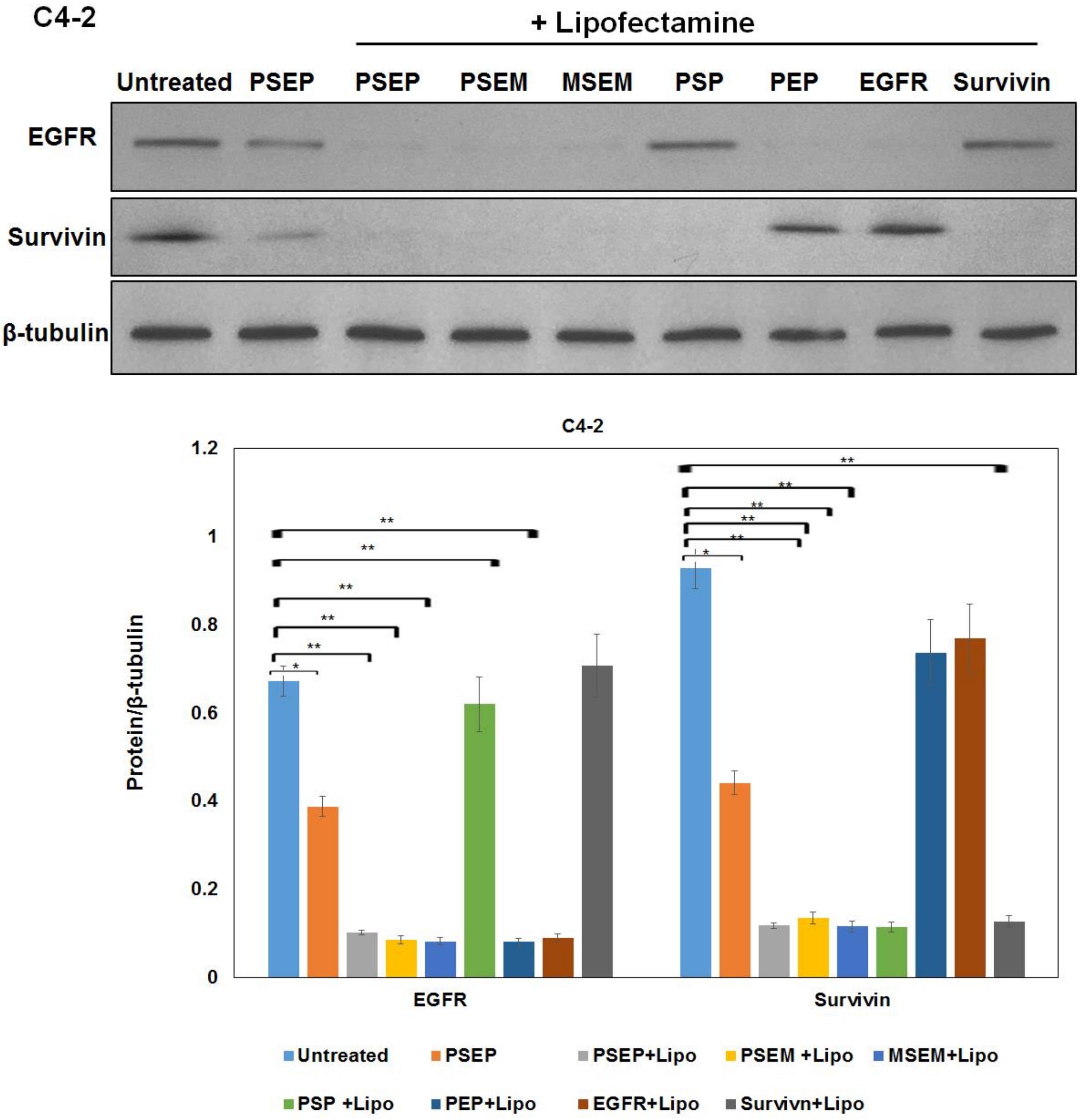

Supplementary Figure 7. Cytotoxicity of chimeras or siRNAs transfected with lipofectamine RNAiMAX in C4-2 cells. Controls are untreated cells or 2'-F- PSEP treatment without lipofectamine. The constructs transfected with lipofectamine are native and have no 2'-fluorine label. C4-2 cells were treated with 100nM each construct for 72 h. (a) Detection of knockdown of EGFR and survivin by Western blot. (b) Quantification of Western blot. Protein levels are normalized to β-tubulin. Data show mean± SEM (n=3) *P<0.05, **P< 0.01.

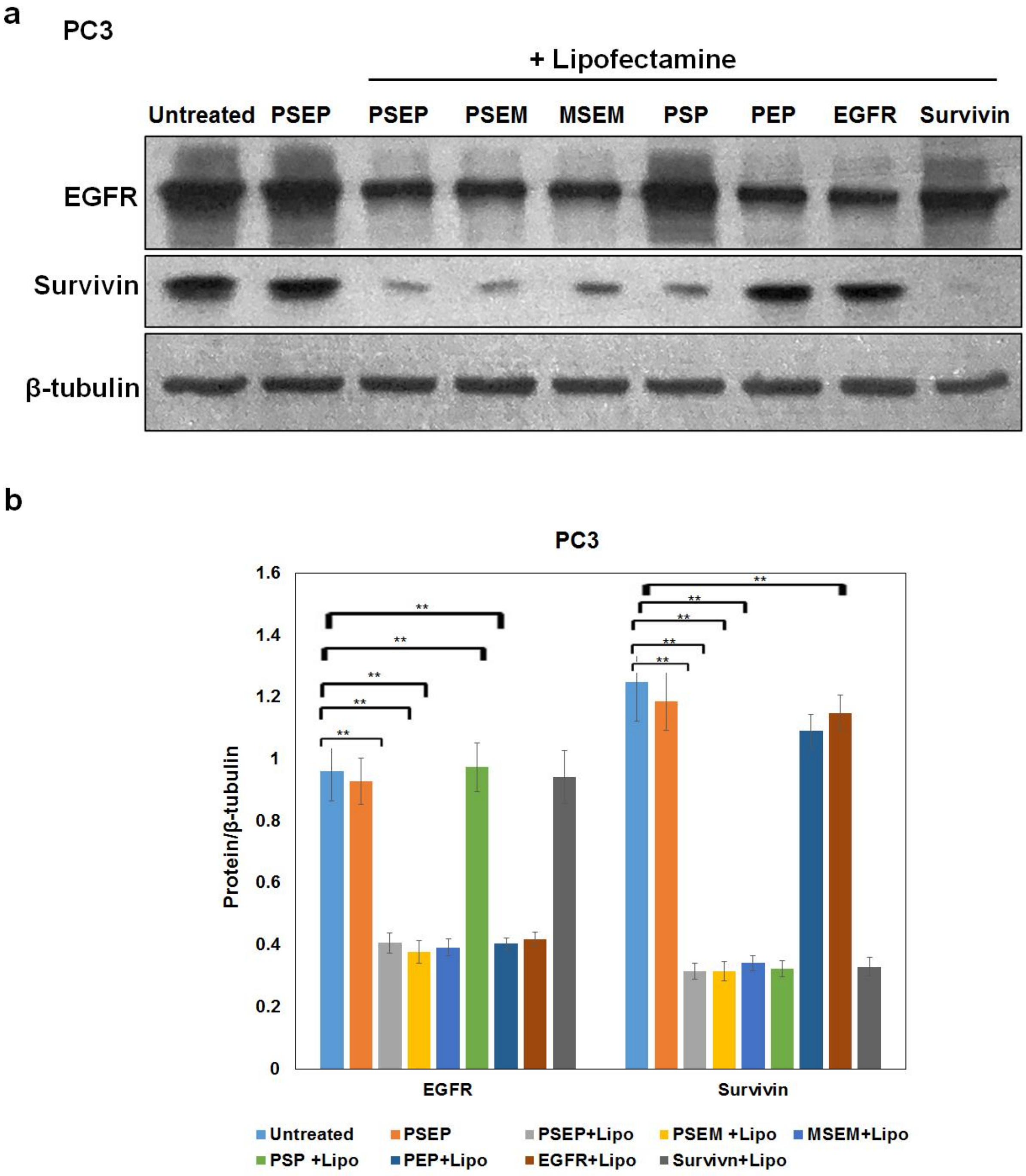

Supplementary Figure 8. Gene silencing efficacy of chimeras or siRNAs transfected with lipofectamine RNAiMAX in PC3 cells. Controls are untreated cells or 2'-F- PSEP treatment without lipofectamine. The constructs transfected with lipofectamine are native and unmodified. PC3 cells were treated with 100nM each construct for 72 h. (a) Detection of knockdown of EGFR and survivin by Western blot. (b) Quantification of Western blot. Protein levels are normalized to β-tubulin. Data show mean± SEM (n=3) *$P<0.05$, **$P< 0.01$.

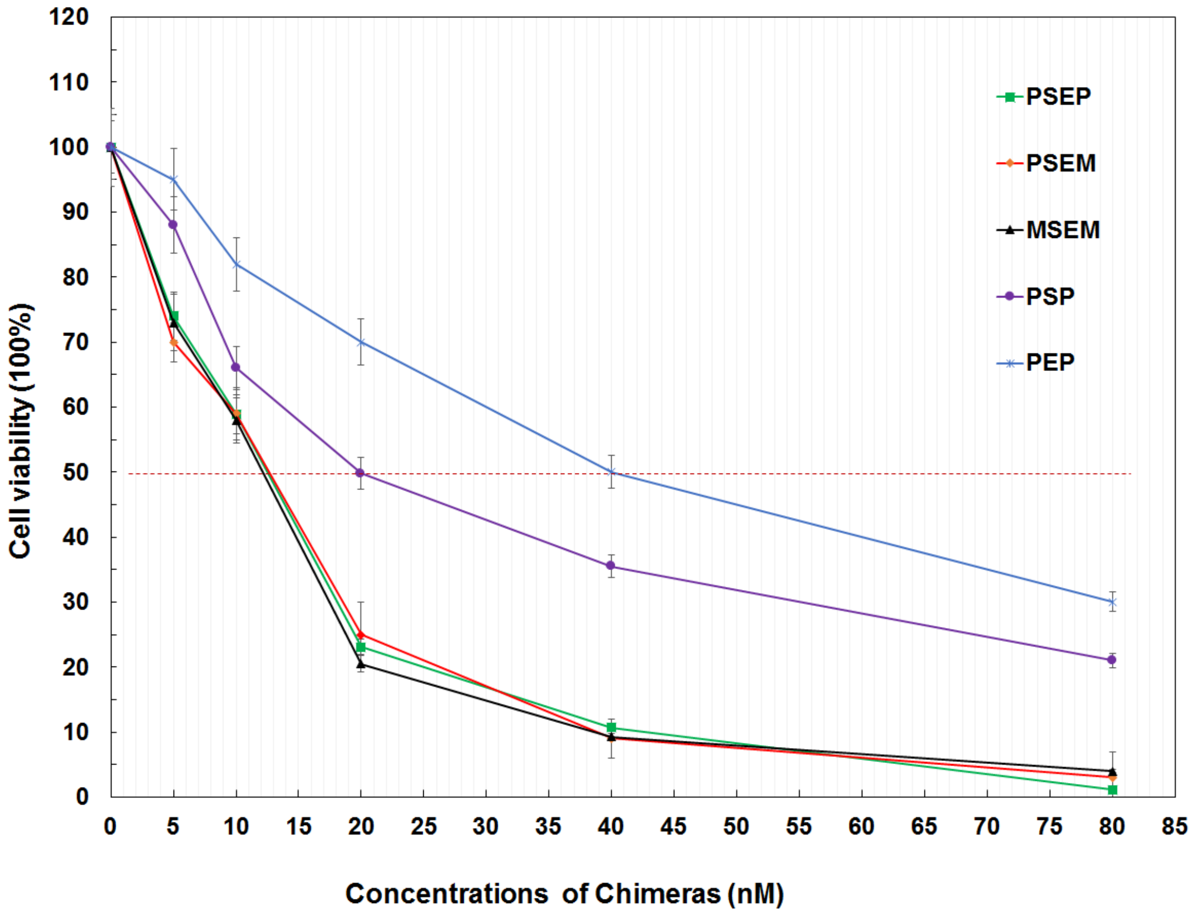

Supplementary Figure 9. Cytotoxicity of chimeras (unmodified) transfected with lipofectamne RNAiMAX. C4-2 cells were treated with varying concentrations of chimeras or siRNAs for 72h. Cell viability was evaluated with CCK-8 agent following the manufacture's instruction. The results are representative of three independent experiments.

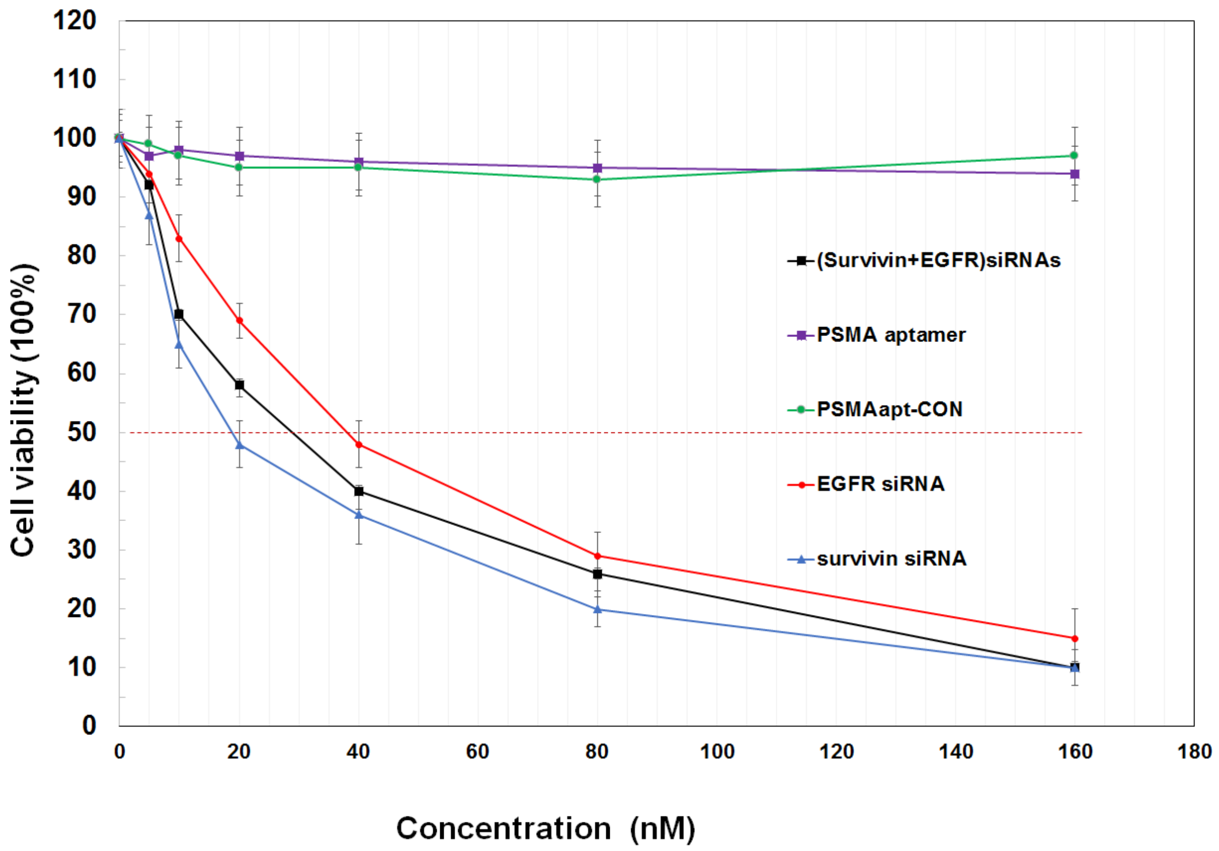

Supplementary Figure 10. Cytotoxicity of PSMA aptamer or siRNA transfected with lipofectamine RNAiMAX. C4-2 cells were treated with varying concentrations of PSMA aptamer (unmodified) or siRNAs (unmodified) for 72h. Cell viability was evaluated with CCK-8 agent following the manufacture's instruction. The results are representative of three independent experiments.

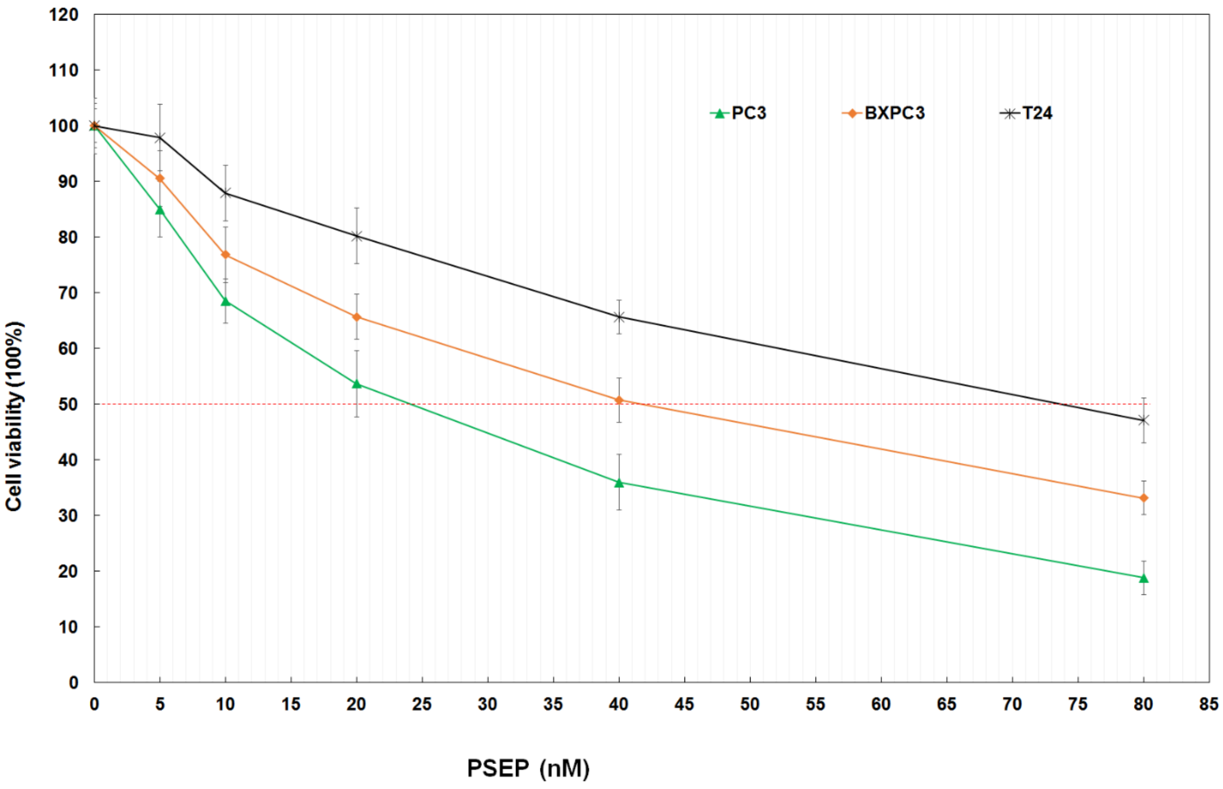

Supplementary Figure 11. Cytotoxicity of PSEP transfected with lipofectamine RNAiMAX in PC3, BXPC3 and T24 cells. PSMA-negative PC3, BXPC3 and T24 cells were treated with varying concentrations of PSEP (unmodified) for 72h. Cell viability was evaluated with CCK-8 agent following the manufacture's instruction. The results are representative of three independent experiments.

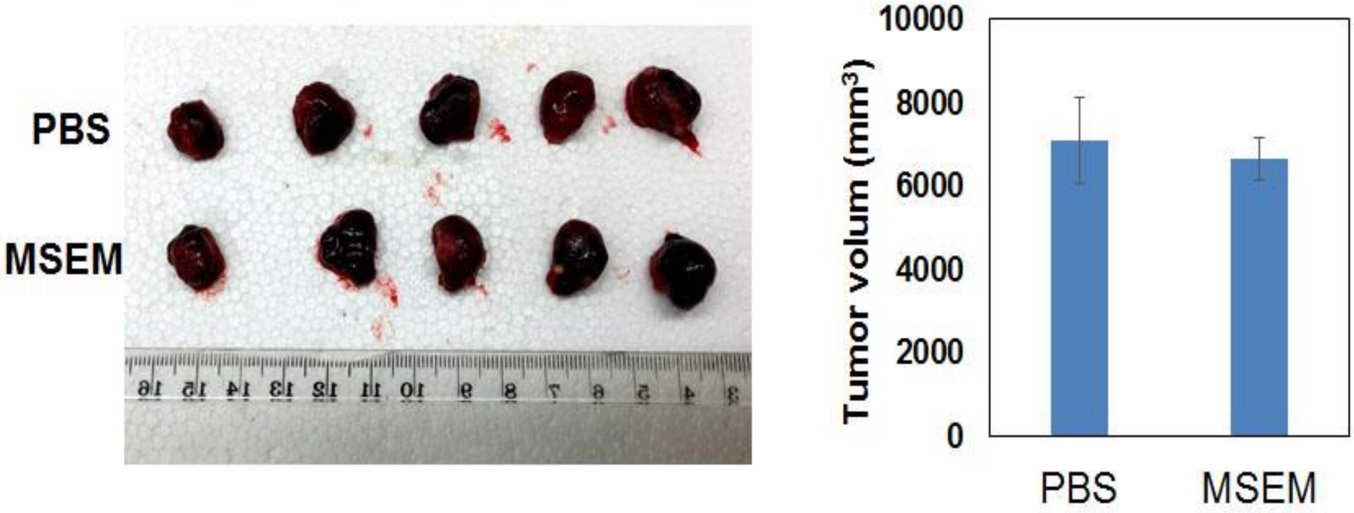

Supplementary Figure 12. Evaluation of treatment efficacy of cell non-targeting MSEM chimera with the same size as PSEP. Athymic nu/nu mice were injected subcutaneously with C4-2 cells mixed with matrigel (v/v 1:1). MSEM (100µl, 20µM) or PBS (100µl) was intraperitoneally injected into the mice every other day for 7 days and followed by injection every day for 14 days. Through measuring tumor size, no significant efficacy was observed upon the treatment with MSEM chimera.

a

Standard		Mouse sera	
IFNα (pg/ml)	OD450	Groups	OD450
0	0.154±0.013	PBS	0.152±0.022
12.5	0.212±0.033	PSEP	0.148±0.012
25	0.325±0.041		
50	0.441±0.021		
100	0.932±0.054		

b

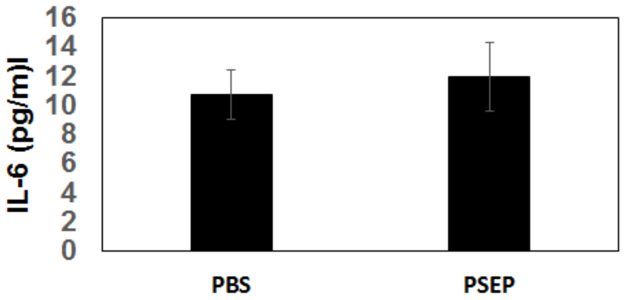

Supplementary Figure 13. Detection of IFNα and IL-6 in sera of mice treated with PBS or PSEP by ELISA. At the endpoint of treatment, blood was collected from facial vein and serum was separated by spin. IFNα was detected with mouse IFN alpha and IL-6 ELISA kits. (a) IFNα; (b) IL-6. No statistically differences were identified for IFNα and IL-6 between PBS and PSEP groups.

.

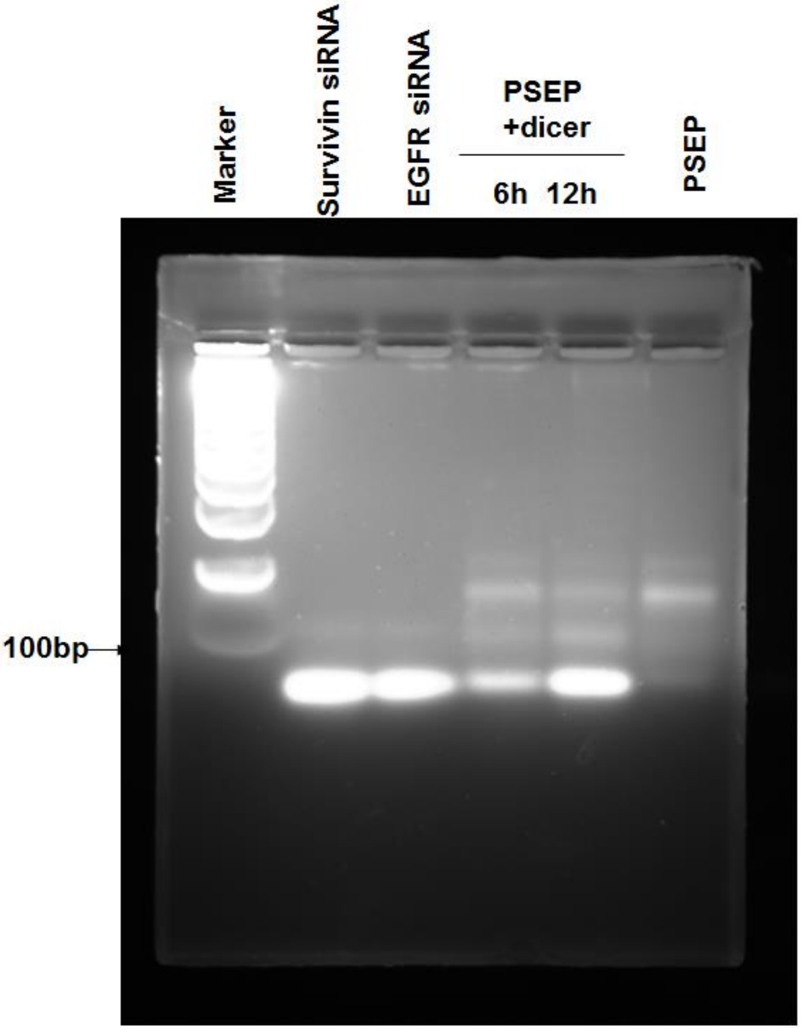

Supplementary Figure 14. Full length gel of Figure1c.

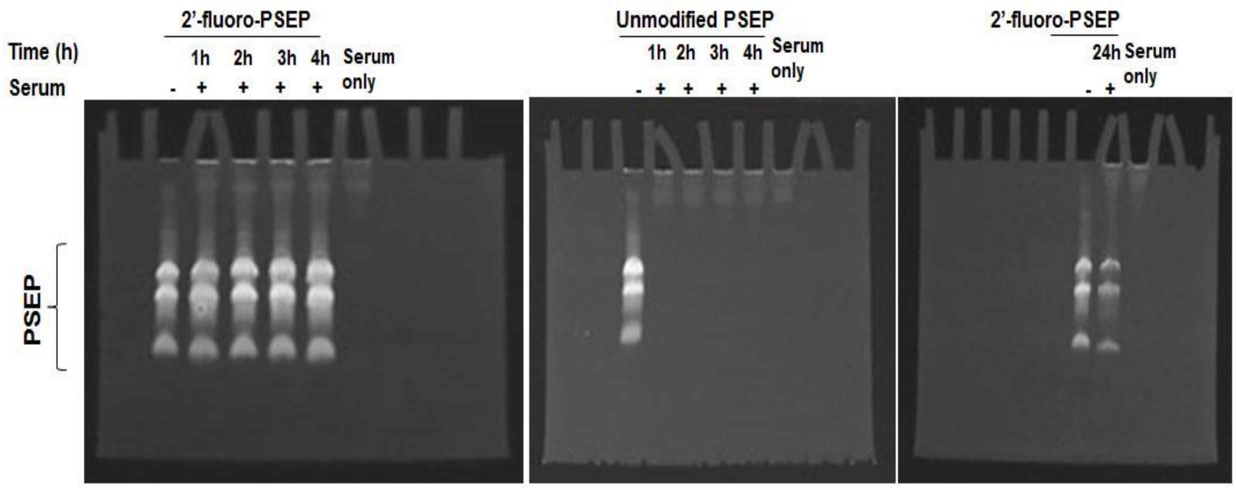

Supplementary Figure 15. Full length gels of Figure 1d.

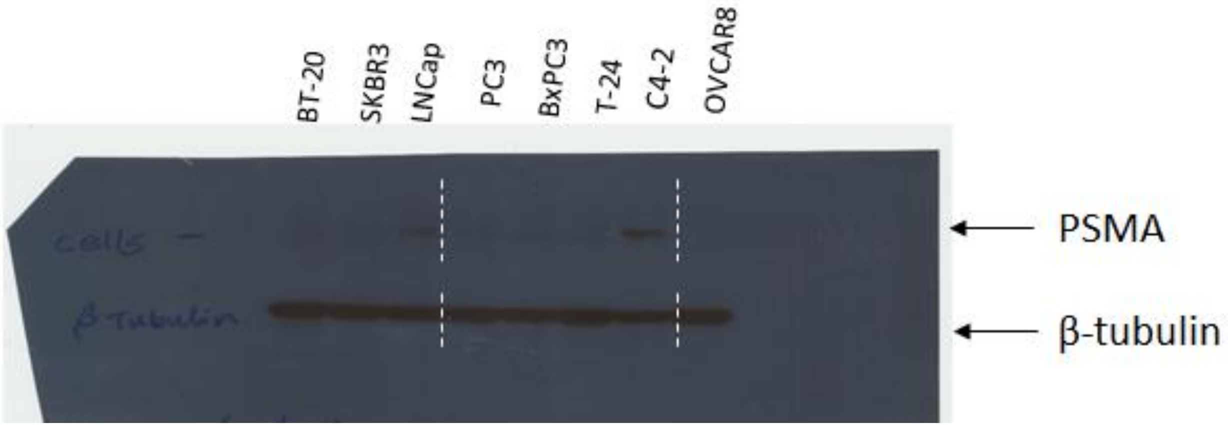

Supplementary Figure 16. The scanned full blot of Figure2a. White dotted lines show the cropping locations. Brightness was adjusted during processing this blot.

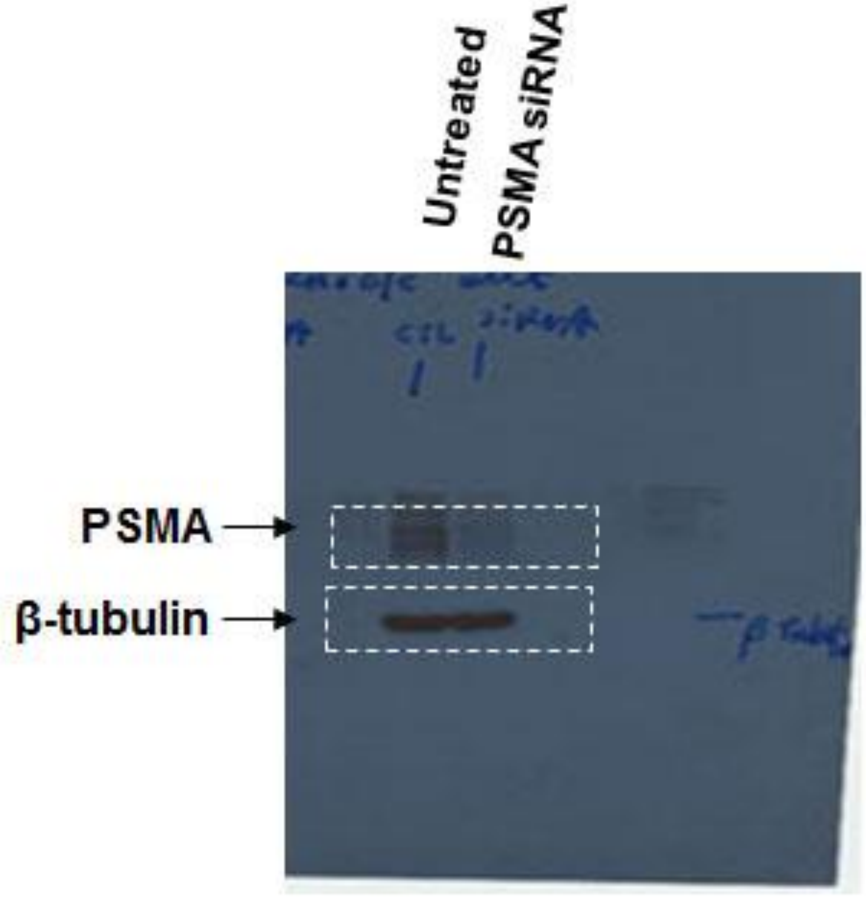

Supplementary Figure 17. The scanned full blots of Figure2b. White dotted lines show the cropping locations

.

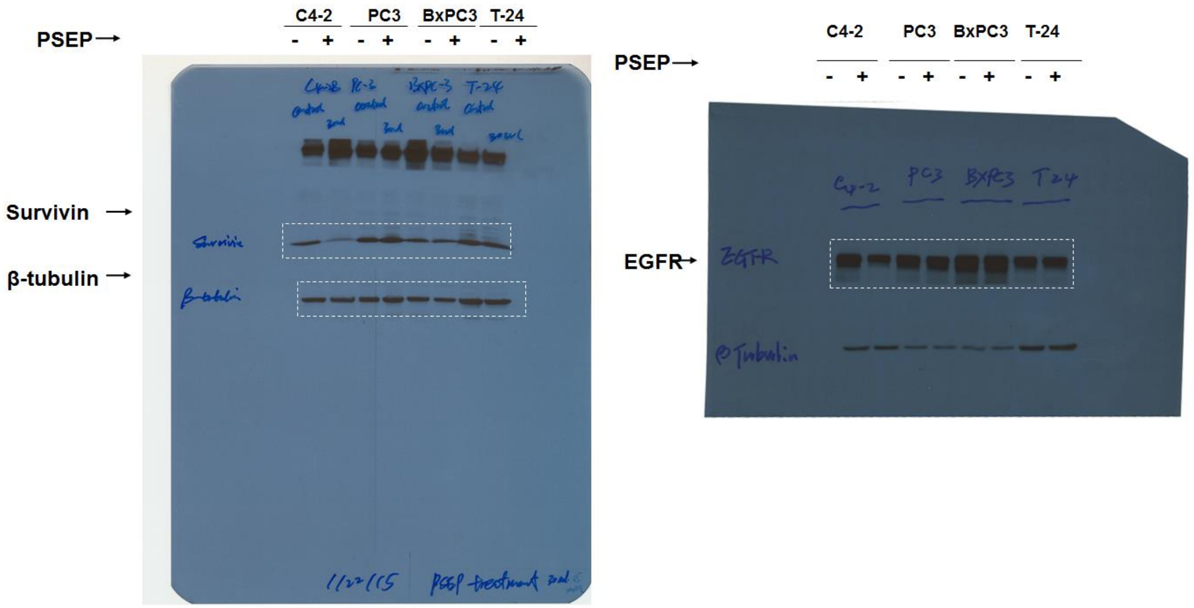

Supplementary Figure 18. **The scanned full blots of Figure 2d.** White dotted lines show the cropping locations.

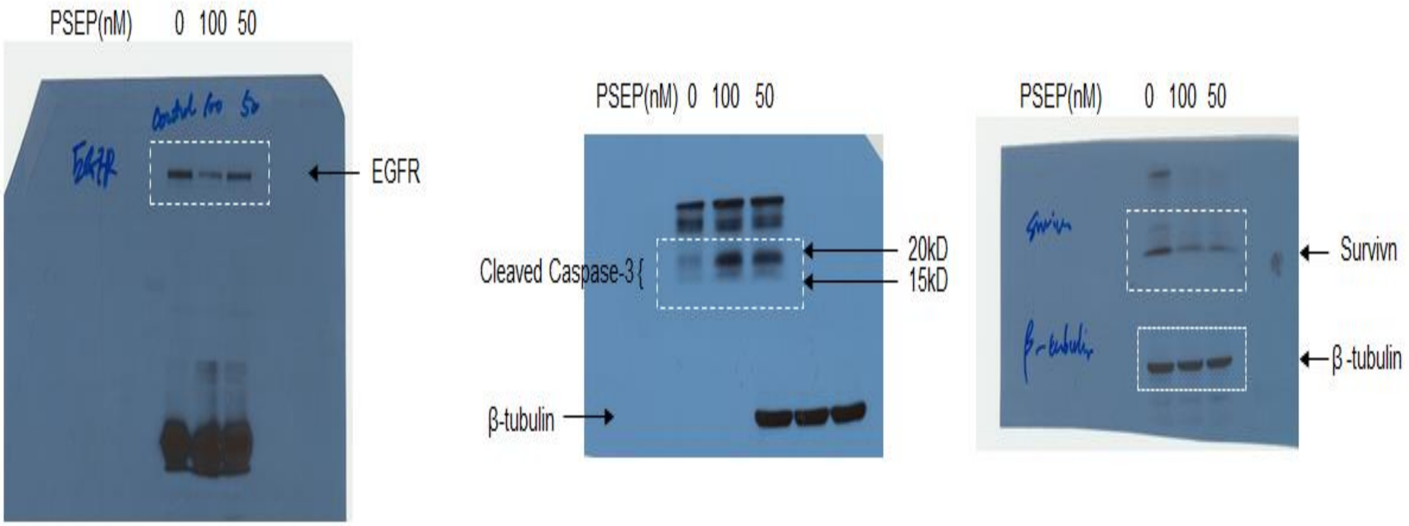

Supplementary Figure 19. The scanned full blots of Figure 2f. White dotted lines indicate the cropping locations

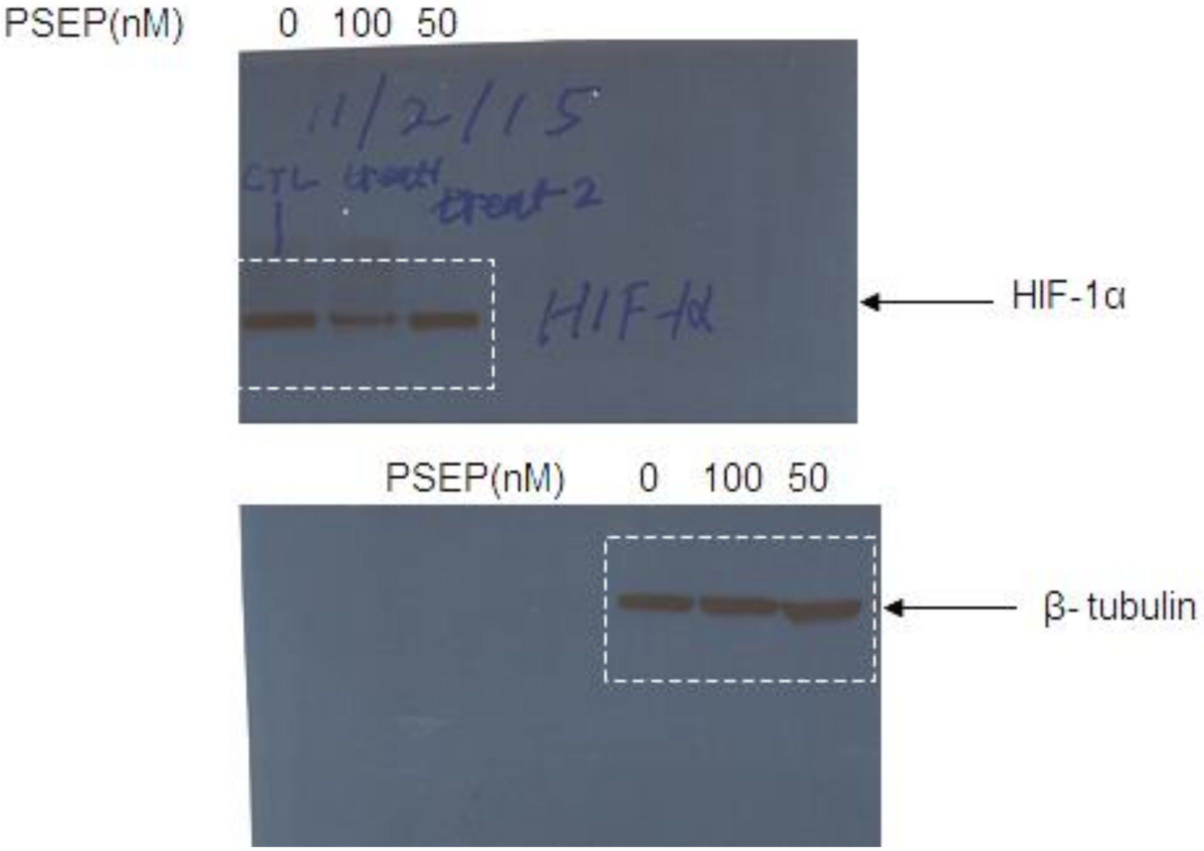

Supplementary Figure 20. The scanned full blots of Figure 5c. White dotted lines show the cropping locations. Brightness was adjusted during processing these blots.

.

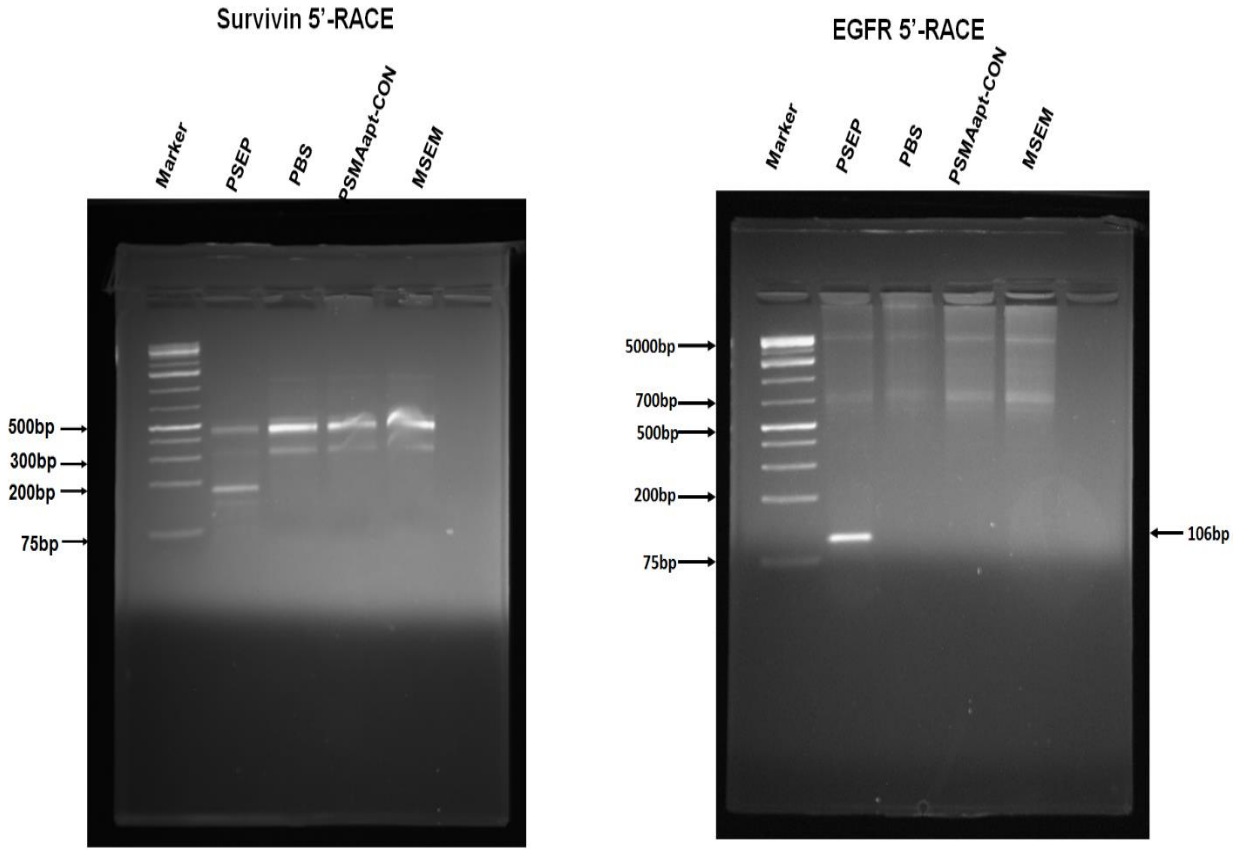

Supplementary Figure 21. Full length gel of Figure 8.
